# Resolving the full spectrum of human genome variation using Linked-Reads

**DOI:** 10.1101/gr.234443.118

**Published:** 2019-04

**Authors:** Patrick Marks, Sarah Garcia, Alvaro Martinez Barrio, Kamila Belhocine, Jorge Bernate, Rajiv Bharadwaj, Keith Bjornson, Claudia Catalanotti, Josh Delaney, Adrian Fehr, Ian T. Fiddes, Brendan Galvin, Haynes Heaton, Jill Herschleb, Christopher Hindson, Esty Holt, Cassandra B. Jabara, Susanna Jett, Nikka Keivanfar, Sofia Kyriazopoulou-Panagiotopoulou, Monkol Lek, Bill Lin, Adam Lowe, Shazia Mahamdallie, Shamoni Maheshwari, Tony Makarewicz, Jamie Marshall, Francesca Meschi, Christopher J. O'Keefe, Heather Ordonez, Pranav Patel, Andrew Price, Ariel Royall, Elise Ruark, Sheila Seal, Michael Schnall-Levin, Preyas Shah, David Stafford, Stephen Williams, Indira Wu, Andrew Wei Xu, Nazneen Rahman, Daniel MacArthur, Deanna M. Church

**Affiliations:** 1 10x Genomics, Pleasanton, California 94566, USA;; 2 The Institute of Cancer Research, Division of Genetics and Epidemiology, London SM2 5NG, United Kingdom;; 3 Analytic and Translational Genetics Unit, Massachusetts General Hospital, Boston, Massachusetts 02114, USA;; 4 Program in Medical and Population Genetics, Broad Institute of MIT and Harvard, Cambridge, Massachusetts 02142, USA

## Abstract

Large-scale population analyses coupled with advances in technology have demonstrated that the human genome is more diverse than originally thought. To date, this diversity has largely been uncovered using short-read whole-genome sequencing. However, these short-read approaches fail to give a complete picture of a genome. They struggle to identify structural events, cannot access repetitive regions, and fail to resolve the human genome into haplotypes. Here, we describe an approach that retains long range information while maintaining the advantages of short reads. Starting from ∼1 ng of high molecular weight DNA, we produce barcoded short-read libraries. Novel informatic approaches allow for the barcoded short reads to be associated with their original long molecules producing a novel data type known as “Linked-Reads”. This approach allows for simultaneous detection of small and large variants from a single library. In this manuscript, we show the advantages of Linked-Reads over standard short-read approaches for reference-based analysis. Linked-Reads allow mapping to 38 Mb of sequence not accessible to short reads, adding sequence in 423 difficult-to-sequence genes including disease-relevant genes *STRC*, *SMN1*, and *SMN2*. Both Linked-Read whole-genome and whole-exome sequencing identify complex structural variations, including balanced events and single exon deletions and duplications. Further, Linked-Reads extend the region of high-confidence calls by 68.9 Mb. The data presented here show that Linked-Reads provide a scalable approach for comprehensive genome analysis that is not possible using short reads alone.

Since the completion of the human genome project, many studies have applied whole-genome sequencing to thousands of individuals from diverse populations, reshaping our understanding of human variation (The 1000 Genomes Project Consortium 2015; Sudmant et al. 2015; Lek et al. 2016). To date, most genome analyses were performed with short reads, resulting in robust analyses of small variants over nonrepetitive parts of the genome. However, recent technical advances in both sequencing and mapping have revealed that despite extensive information garnered from short-read large population surveys, we are still underrepresenting the amount of structural variation in the human population (Chaisson et al. 2015, 2017; Huddleston and Eichler 2016; Collins et al. 2017).

The reconstruction of haplotypes (phasing) can be important for many biological studies but is currently not feasible for single samples sequenced with short reads. When analyzing data from rare disease cohorts, knowing if potentially pathogenic variants are in *cis* or *trans* is necessary for interpreting clinical impact. In addition, haplotype information is necessary for understanding allele-specific impacts on gene expression (Ramaker et al. 2017). Studies also show that haplotype information can be critical for variant identification, particularly for heterozygous SVs (Huddleston and Eichler 2016).

The limitations of short reads suggest the need for improved methods for genome analysis. Several long-molecule sequencing and mapping approaches have been developed (Carneiro et al. 2012; Bionano Genomics 2017; Nakano et al. 2017), but their high input requirements, error rates, and costs make them intractable for many applications, particularly those requiring thousands of samples (Chaisson et al. 2017). To address this need, we developed a technology that retains long range information while maintaining the benefits of short-read sequencing (Zheng et al. 2016). The core data type, Linked-Reads, is generated by performing haplotype limiting dilution of long DNA molecules into more than 1 million barcoded partitions, synthesizing barcoded sequence libraries within those partitions, and then performing standard short-read sequencing in bulk. The limited amount of DNA put into the system, coupled with novel algorithms, allow short reads to be associated with their long molecule of origin, in most cases, with high probability.

Here, we describe both biochemistry and algorithmic improvements over the original Linked-Reads platform, GemCode, using the Chromium System. It is important to note that Linked-Reads are paired-end short reads with a barcode on read 1 and can be used by many common short-read tools. To fully realize the potential of Linked-Reads, additional algorithms that take advantage of these barcoded sequences and molecule information must be combined with short-read algorithms. In the following text, when we refer to Linked-Read WGS (lrWGS) we are referring to the combination of biochemistry and algorithm approaches applied. We use srWGS (short-read whole genome sequencing) and srWES (short-read whole exome sequencing) to refer to whole-genome and whole-exome results from Illumina TruSeq PCR-free processed with a GATK best practices pipeline, as described subsequently.

## Results

### Improvements in Linked-Read data

**Figure 1.**
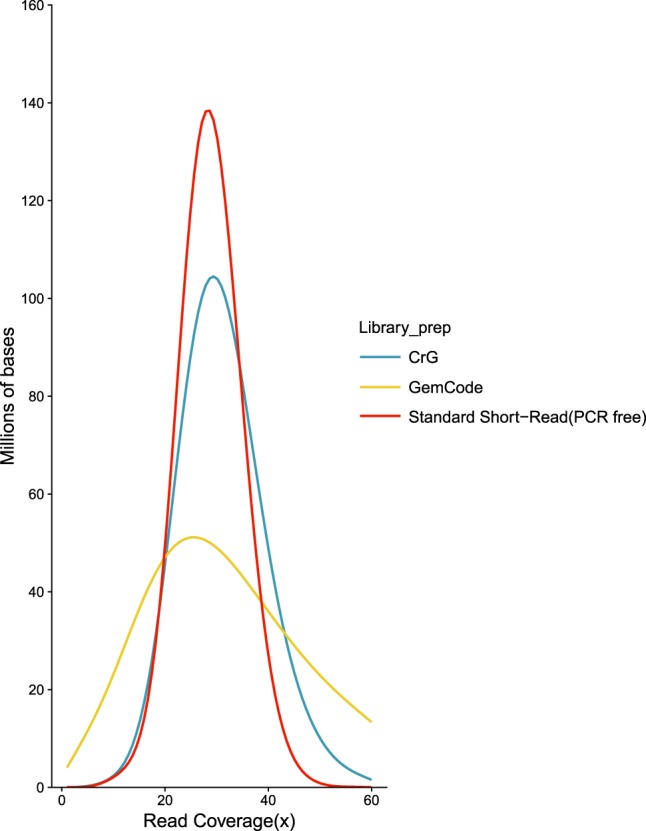
Coverage evenness. Distribution of read coverage for the entire human genome (GRCh37). Comparisons between 10x Genomics Chromium Genome (CrG), 10x Genomics GemCode (GemCode), and Illumina TruSeq PCR-free standard short-read NGS library preparations (Standard Short-Read [PCR-Free]). Sequencing was performed in an effort to match coverage (Methods). Note the shift of the CrG curve to the *right*, showing the improved coverage of Chromium versus GemCode. The *x*-axis represents the fold read coverage across the genome, and the *y*-axis represents the total number of bases covered at any given read depth.

One limitation of the original GemCode approach was the need to combine the Linked-Read data with a standard short-read library due to coverage imbalances in the GemCode library. By modifying the biochemistry to include isothermal amplification, we were able to obtain more even genome coverage, approaching that of PCR-free short-read preparations and eliminating the need for an additional library (Fig. 1).

Additional improvements include increasing the number of barcodes from 737,000 to 4,000,000 and the number of partitions from 100,000 to more than 1,000,000. This allows for fewer DNA molecules per partition and thus greatly reduces the rate at which two allelic loci occur in the same GEM (Supplemental Fig. 1). This lowered rate of barcode sharing increases the probability of correctly associating a short read to its molecule of origin.

### Improved genome and exome alignments

**Figure 2.**
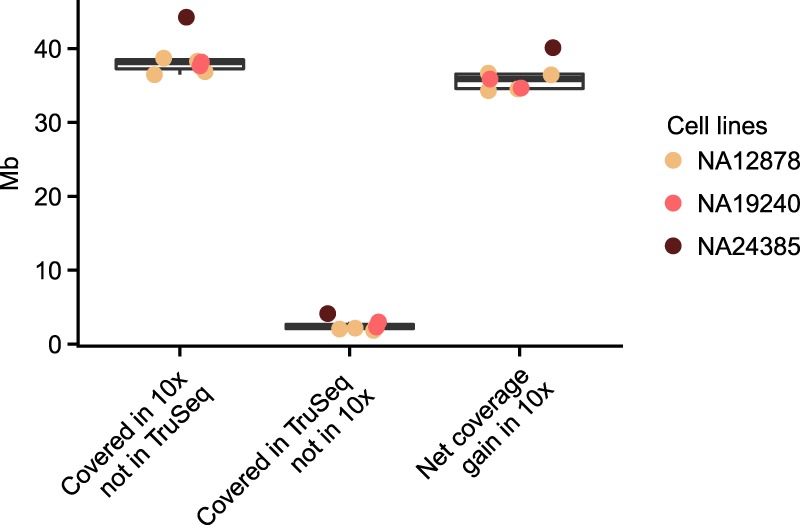
Comparison of unique genome coverage by assay. The *y*-axis shows the amount of sequence with a coverage of ≥5 reads at MapQ ≥30. Column 1 shows amount of the genome covered by 10x Chromium where PCR-free TruSeq does not meet that metric. Column 2 shows the amount of the genome covered by PCR-free TruSeq where 10x Chromium does not meet the metric. Column 3 shows the net gain of genome sequence with high-quality alignments when using 10x Chromium versus PCR-free TruSeq. The comparison was performed on samples with matched sequence coverage (Methods).

Several improvements were made in the Long Ranger analysis pipeline to better take advantage of the Linked-Read data type. The first of these, the Lariat aligner (https://github.com/10XGenomics/lariat), expands on the “Read-Cloud” approach (Bishara et al. 2015; Supplemental Methods 1). This approach allows for the recovery of 36–44 Mb of genome coverage when compared to PCR-free short reads. Conversely, only 1–4 Mb of the genome has coverage in the PCR-free data but not lrWGS (Fig. 2). The amount of recovered alignments using lrWGS varies from chromosome to chromosome, but is consistent across samples (Supplemental Fig. 2). The ability of lrWGS + Lariat to rescue repetitive sequence depends on repeat elements being far enough from each other that they are not likely to share a barcode, and repeat type and distribution differs by chromosome. The sequence gained using lrWGS is dominated by regions annotated as segmental duplications (∼75%), with the alignments to the decoy sequence accounting for another 13% and exonic sequences accounting for ∼5% (Fig. 2; Supplemental Methods 1.2; Supplemental Table 1). Input molecule length also impacts the amount of sequence recovered (Supplemental Fig. 3).

**Figure 3.**
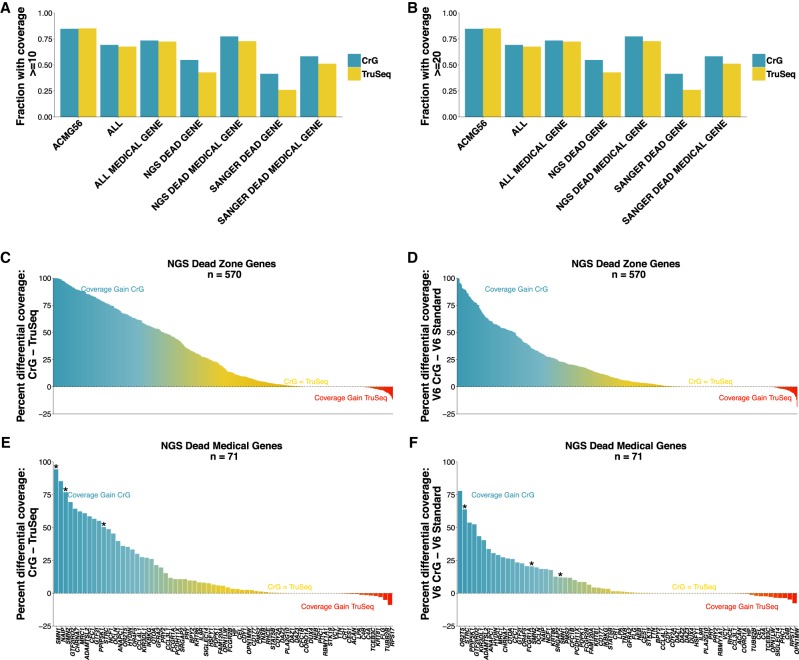
Gene finishing metrics. Gene finishing metrics for whole-genome and whole-exome sequencing across selected gene sets. Genome is shown on *left*, exome on *right*. Gene finishing is defined as the percentage of exonic bases with at least 10-fold coverage for genome (*A*) and at least 20× for exome (*B*) (Mapping quality score ≥MapQ30). (*A*,*B*) Gene finishing statistics for seven disease-relevant gene panels. Shown is the average value across all genes in each panel. Although Chromium provides a coverage advantage in all panel sets, the impact is particularly profound for “NGS Dead Zone” genes. (*C*–*F*) Net coverage differences for individual genes when comparing Chromium to PCR-free TruSeq. Each bar shows the difference between the coverage in PCR-free TruSeq from the coverage in 10x Chromium. (*C*,*D*) The 570 NGS “dead zone” genes for genome (*C*) and exome (*D*). (*E*,*F*) The graphs are limited to the list of NGS dead zone genes implicated in Mendelian disease. In *C*–*F*, the blue coloring highlights genes that are inaccessible to short-read approaches, but accessible using CrG; the yellow coloring indicates genes where CrG is equivalent to short reads or provides only modest improvement. The red coloring shows genes with a slight coverage increase in TruSeq, although these genes are typically still accessible to CrG. (*) Genes *SMN1*, *SMN2*, and *STRC*. The comparison was performed on samples with matched coverage (Methods).

We observe a net gain in gene coverage when performing lrWGS compared to srWGS, and even more robust improvement when performing lrWES compared to srWES (Supplemental Fig. 4). In a known set of 570 genes with closely related paralogs that confound short-read alignment (NGS “dead zone” genes) (Mandelker et al. 2016), we see a net gain in read coverage in 423 genes using lrWGS and 376 using lrWES. For the 71 NGS “dead zone” genes relevant to Mendelian disease, we see a net improvement in 51 of these genes using lrWGS and 41 genes using lrWES (Fig. 3).

### Small variant calling

Next, we assessed the performance of Linked-Reads for small variant calling (<50 bp). We used control samples, NA12878 and NA24385 as test cases. We produced two small variant call sets for each sample, one generated by running paired-end 10x Linked-Read Chromium libraries through the Long Ranger (lrWGS) pipeline and one produced by analyzing paired-end reads from a PCR-free TruSeq library using GATK pipeline (PCR-) following best practices recommendations (https://software.broadinstitute.org/gatk/best-practices/). The number of calls was comparable between data sets and were largely overlapping (Table 1).

**Table 1.**
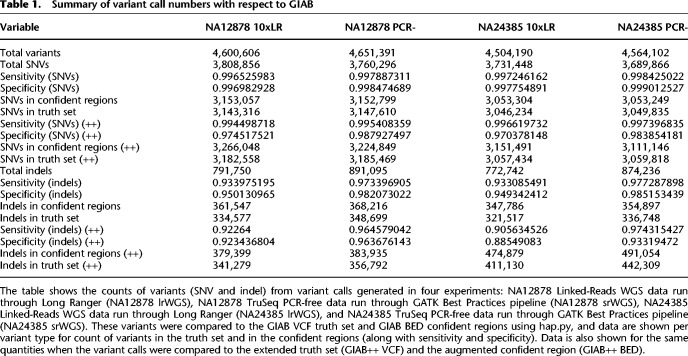
Summary of variant call numbers with respect to GIAB

In order to assess the accuracy of the variant calling in each data set, we used the hap.py tool (https://github.com/Illumina/hap.py, commit 6c907ce) to compare the lrWGS and PCR- VCFs to the Genome in a Bottle (GIAB) high-confidence call set (v. 3.2.2) (Zook et al. 2014). We chose this call set version as it was the last GIAB data set that did not include 10x data as an input for call set curation. This necessitated the use of GRCh37 as a reference assembly rather than the more current GRCh38 reference assembly, limiting analysis to the 82.67% of SNV calls that overlap high-confidence regions. Initial results suggested that the lrWGS calls had comparable sensitivity and specificity for SNVs (Table 1; Supplemental Table 2). We observed slightly diminished indel sensitivity and specificity, driven largely by regions with extreme GC content and low complexity sequences (LCRs).

The GIAB high-confidence data set is known to be conservative, so we explored whether there was evidence for variants called outside of the GIAB set. We utilized publicly available 40-fold coverage Pacific Biosciences (PacBio) data sets available from the GIAB consortium (Zook et al. 2016) and PCR- short-read data to evaluate Linked-Read putative false positive variant calls. Initial manual inspection of 25 random locations suggested that roughly half of the hap.py identified lrWGS false positive calls were well supported by short-read or PacBio evidence and were haplotype consistent (Supplemental Table 3). We then did a global analysis of all 9513 SNVs and 18,030 indel putative false positive calls identified in NA12878 and looked for evidence of the alternate alleles in aligned PacBio reads only. This analysis provided evidence that 2377 SNVs and 12,812 indels of the GIAB determined false positive calls were likely valid calls (Supplemental Fig. 5; Supplemental File 1). This prompted us to extend our analysis to include 69.72 Mb for NA12878 and 70.66 Mb for NA24385 of the genome in addition to the GIAB-defined confident regions (for details on GIAB++ BED, see Methods). We reanalyzed the variant calls with the hap.py tool with the augmented confident regions. This allowed us to identify an additional 19,688 SNVs and 5444 indels as likely true positives. We anticipate that this is a conservative estimate since our hap.py-defined false positive calls are inflated due to lack of PacBio coverage in many of these regions. Of the total putative false positive calls exclusive to the GIAB++ analysis, 61.95% (45,665) of SNVs and 42.08% (4637) of indels could not be validated because of little or no PacBio read coverage (Supplemental Fig. 5). These data show that the lrWGS approach identifies more small variants than short-read only approaches, driven by an increase in the percentage of the genome for which lrWGS can obtain high-quality alignments (Table 1).

### Haplotype reconstruction and phasing

**Figure 4.**
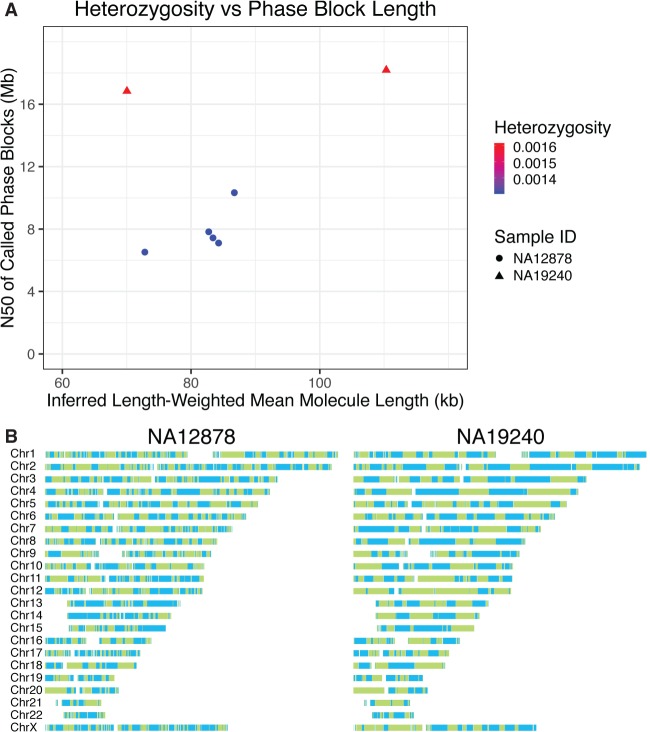
Haplotype reconstruction and phasing. (*A*) Inferred length-weighted mean molecule length plotted against N50 of called Phase blocks (both metrics reported by Long Ranger) and differentiated by sample ID and heterozygosity. Heterozygosity was calculated by dividing the total number of heterozygous positions called by Long Ranger by the total number of non-N bases in the reference genome (GRCh37). Two replicates of NA19240 and five replicates of NA12878 were used. Samples with higher heterozygosity produce longer phase blocks than samples with less diversity when controlling for input molecule length. (*B*) Phase block distributions across the genome for input length matched Chromium Genome samples NA12878 and NA19240. Phase blocks are shown as displayed in Loupe Genome Browser. Solid colors indicate phase blocks. Note the longer phase blocks in the more diverse NA19240 sample.

An advantage of Linked-Reads is the ability to reconstruct multi-megabase haplotypes (phase blocks) from genome sequence data for a single sample. Haplotype reconstruction increases sensitivity for calling heterozygous variants, particularly SVs (Huddleston et al. 2016). It also improves variant interpretation by providing information on the physical relationship of variants, such as whether variants within the same gene are in *cis* or *trans*. In the control samples analyzed, we see phase block N50 values for lrWGS of 10.3 Mb for NA12878, 9.58 Mb for NA24385, 16.8 Mb for NA19240, and 302 kb for lrWES using Agilent SureSelect v6 baits on NA12878. This allowed for complete phasing of 91.1% of genes in the NA12878 genome, 90.9% in NA24385, and 91.0% in NA19240, and an average of 91% in the NA12878 exome. Phase block length is a function of input molecule length, molecule size distribution, and of sample heterozygosity extent and distribution. At equivalent mean molecule lengths, phase blocks will be longer in more diverse samples (Fig. 4; Supplemental Fig. 6). For samples with similar heterozygosity, longer input molecules will increase phase block lengths (Supplemental Fig. 7).

We assessed the accuracy of our phasing calls by comparing the Linked-Read phasing results for NA12878 with the Illumina Platinum genomes (Eberle et al. 2017) phasing results derived from jointly phasing the 17-member CEPH pedigree. Following this analysis (Amini et al. 2014), we decompose phasing errors into “short-switches,” small numbers of isolated variants incorrectly phased, and “long-switches,” errors in which an incorrect phasing junction persists for many variants across a longer distance. The rate of each switch type is measured per phased heterozygous variant. We also measure (1) the rate at which a SNP is correctly phased to other variants in its phase block (heavily penalizing long switch errors inside large phase blocks), and (2) the rate at which a SNP inside a gene is correctly phased to other variants in the gene. Independent studies have demonstrated that Linked-Read phasing has best-in-class accuracy compared to a variety of other phasing methods (Chaisson et al. 2017; Choi et al. 2018). Short switch error rates average ∼2 × 10^−4^, long switch error rates average ∼2 × 10^−5^, and within-phase-block correct rate of ∼0.98 (Supplemental Table 4).

Phase block construction using lrWES is, in addition, constrained by the capture bait set and reduced variation in coding sequences. In order to analyze additional factors impacting phase block construction, we assessed four samples with known compound heterozygous variants in three Mendelian disease genes, *DYSF*, *POMT2*, and *TTN*. The variant separation ranged from 33 to >188 kb (Table 2). Initial DNA extractions yielded long molecules ranging in mean size from 75 to 112 kb. We analyzed these samples using the Agilent SureSelect V6 exome bait set, with down-sampling of sequence data to both 7.25 Gb (∼60-fold coverage) and 12 Gb of sequence (∼100-fold coverage). In all cases, the variants were phased with respect to each other and determined to be in *trans*, as previously determined by orthogonal assays. By comparing phasing of NA12878 Linked-Read exome data to phasing from pedigree analysis of the Illumina Platinum Genomes CEPH pedigree, we determine that the global probability a SNP is phased correctly within a gene ranges from 99.95–99.99%, making misphasing of two heterozygous variants in a gene relative to each other a very rare event.

**Table 2.**
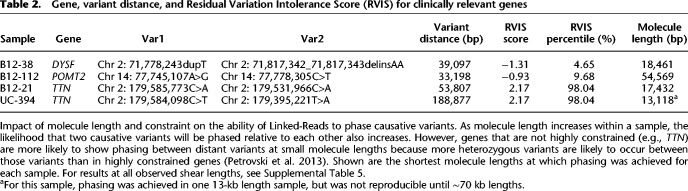
Gene, variant distance, and Residual Variation Intolerance Score (RVIS) for clinically relevant genes

**Figure 5.**
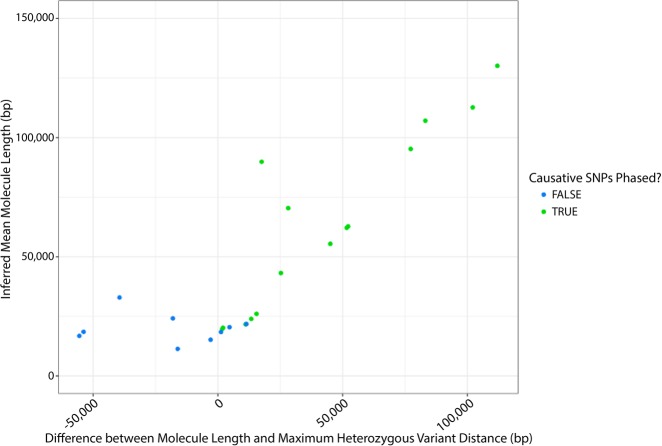
Validated example of impact of molecule length on phasing (7.25 Gb). Blue dots represent samples for which the variants of interest are not phased, and green dots represent samples for which there is phasing of the variants of interest. At longer molecule lengths (>50 kb), the molecule length was always longer than the maximum distance between heterozygous SNPs in a gene, and phasing between the causative SNPs was always observed. As molecule length shortens, it becomes more likely that the maximum distance between SNPs exceeds the molecule length (reflected as a negative difference value), and phasing between the causative SNPs was never observed in these cases. When maximum distance is similar to the molecule length, causative SNPs may or may not be phased. This is likely impacted by the molecule length and variant distribution within the sample.

Many samples of interest have already been extracted using standard methods not optimized for high molecular weight DNA and may not be available for a fresh reextraction. For this reason, we wanted to understand the impact of reduced molecule length on phasing of genes and variants in these samples. We took freshly extracted long molecules and sheared them to various sizes, aiming to assess lengths ranging from 5 kb to the original full length (Table 2; Supplemental Table 5). These results illustrate the complex interplay between molecule length distribution and observed heterozygosity within a region. For example, in sample B12-21, with variants in *TTN* that are 53 kb apart, the variants could be phased, even with the smallest molecule size. However in sample B12-122, with variants in *POMT2* only 33 kb apart, variant phasing is lost at 20-kb lengths. This appeared to be due to a higher rate of heterozygous variation in *TTN*, allowing the phasing of distant heterozygous sites to occur by phasing the many other heterozygous variants that occurred between them. A general lack of variation in *POMT2* precluded such “stitching” together of shorter molecules by phasing of intermediate heterozygous variation. To confirm this, we assessed the maximum distance between heterozygous sites observed in each gene in each sample. As expected, when the maximum distance between heterozygous SNPs is greater than the molecule length (negative values), the ability to phase causative SNPs decreases (Fig. 5). There are exceptions to this as longer molecules in the size distribution will sometimes allow tiling between variants, extending phase block size beyond what would be expected based on the mean length alone.

Linked-Reads allow for the reconstruction of long haplotypes. Optimizing for long input molecules provides for maximum phase block size, but shorter molecule lengths can provide gene-level phasing. Further, in the context of sequencing for disease identification, causative heterozygous variants would be expected to aid in phasing of the disease-causing gene as they would provide the necessary variation to distinguish the two haplotypes.

### Structural variant detection

Structural variants remain one of the most difficult types of variation to accurately ascertain, in part because they tend to cluster in duplicated and repetitive regions, but also because the various signals for these events can be challenging to detect with short reads. Another complicating factor is that there are many types of structural variants, and each requires the detection of a different signal (Alkan et al. 2011; Collins et al. 2017). There is increasing evidence that grouping reads by their source haplotype improves SV sensitivity, but this is not commonly done in practice (Huddleston et al. 2016; Chaisson et al. 2017).

#### Large-scale SVs (>30 kb)

Long Ranger uses two novel algorithms to identify large SVs—one that assesses deviations from expected barcode coverage, and one that looks for unexpected barcode overlap between distant regions. The barcode coverage algorithm is useful for assessing CNVs, whereas the barcode overlap method can detect a variety of SVs but fails to detect terminal events (Supplemental Section 3). We used two approaches to assess lrWGS performance on large SVs. First, we compared SV calls from the NA12878 sample to validated calls described in a publication of a structural variant classifier, svclassify (Parikh et al. 2016). Next, we obtained the GeT-RM CNVPanel, a collection of known events including large deletions, duplications, inversions, balanced translocations, and unbalanced translocations designed to assess performance of clinical aCGH.

The validated call set published with svclassify (Parikh et al. 2016) contains deletions and insertions, but no balanced events. In contrast, the Long Ranger pipeline output contains deletions, duplications, and balanced events, but Long Ranger does not currently call insertions (Supplemental Table 6).

We first considered deletion variants >30 kb. The svclassify set contains 11 such deletion calls, Long Ranger calls 17 PASS events, and eight events are common to both (Table 3). All of the eight variants in common show Mendelian consistency and breakpoint agreement within the CEU/CEPH trio. Of the three svclassify calls not called by Long Ranger, one is called by Long Ranger as an event <30 kb, one is called but filtered to the candidate list due to overlap with a segmental duplication, and one is an error in the svclassify set relative to GRCh37.p13 (Supplemental Section 4.1). We checked for Mendelian consistency in the nine events unique to the Long Ranger set. Eight of these events showed consistent inheritance, although two had inconsistent breakpoints when compared to the parents (Supplemental Table 7). The last event is a call in the telomeric region of Chr 2 that overlaps a known reference assembly issue. The call appeared to be made due to a drop in phased coverage on one haplotype immediately adjacent to a known reference gap, and is likely a false positive.

**Table 3.**
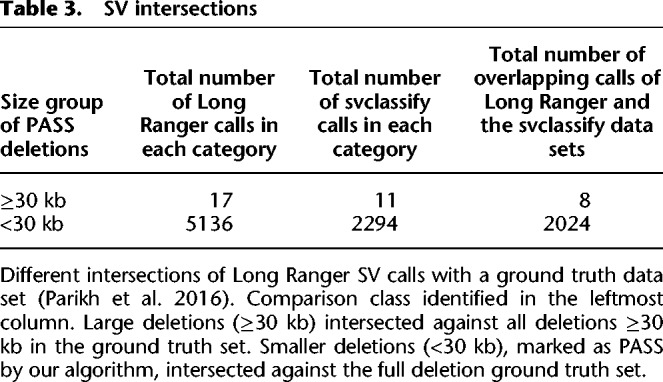
SV intersections

We next tested 23 samples with 29 balanced or unbalanced SVs from the GeT-RM CNVPanel available from Coriell. These samples have multiple, orthogonal assays confirming the presence of their described SVs. We detected 27 of the 29 SVs, correctly characterizing 22 of the 23 samples tested (Supplemental Table 8). One of the undetected events was in the “candidate” SV list as it overlaps a segmental duplication. The missed event is a balanced translocation with a breakpoint in a heterochromatic region of Chr 16. This region is represented by Ns in the reference assembly and will be invisible to any sequence-based reference dependent method (Schneider et al. 2017).

We also assessed the impact of sequence depth on large SV identification in the GeT-RM set. Large CNV signals were detectable with as little as 5 Gb (approximately onefold genomic read coverage) (Supplemental Fig. 8), and balanced events required ∼50 Gb of sequence for the algorithm to call these events, with signal in the data suggesting algorithmic improvements could lessen this requirement (Supplemental Fig. 9).

#### Intermediate SV calls (50 bp–30 kb)

We next considered deletions between 50 bp and 30 kb in NA12878. These deletions were detected both using Long Ranger–specific algorithms as well as the Genome Analysis Toolkit (GATK) HaplotypeCaller. We obtained 1824 deletion calls from GATK and 4118 from Long Ranger (Table 4). These two sets were merged using SURVIVOR (Jeffares et al. 2017) resulting in 5136 merged deletion calls. This compares to 6965 deletions >50 bp per sample in a study combining the output of 13 different algorithms on short-read data and 9488 deletions >50 bp per sample on long-read data (Chaisson et al. 2017). To establish a comparison to existing methods, we ran the LUMPY (Layer et al. 2014) algorithm using the developer recommendations but without tuning parameters (Supplemental Table 9) and found 19,307 deletion calls in this size range.

**Table 4.**
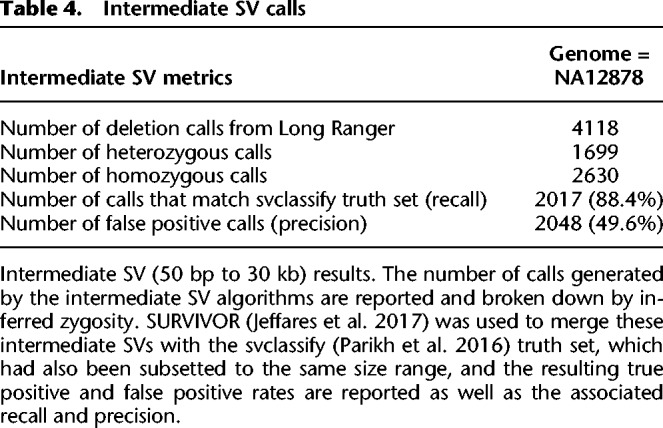
Intermediate SV calls

**Figure 6.**
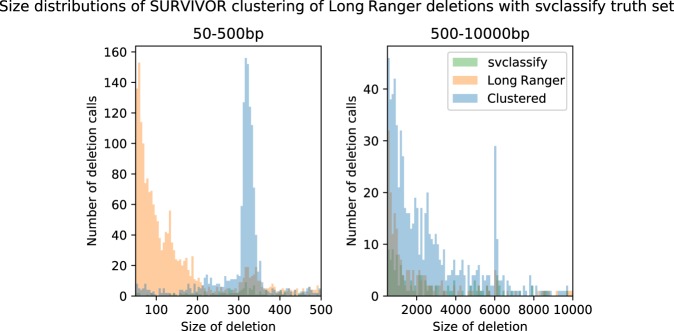
Deletion size distributions. Long Ranger calls intersected with the svclassify truth set by size. True positive calls are blue, false negative calls are green, and false positive calls are orange. Most false positives are present in the <250-bp size range, reflecting the lack of small deletions in the svclassify set. Peaks corresponding to *Alu* and L1/L2 elements can be seen at ∼320 bp and ∼6 kb, respectively.

Using both the output of Long Ranger and LUMPY, we compared our calls to the calls in svclassify. We correctly identified 88.4% of intermediate deletions present in the svclassify truth set (2107), and also called an additional 2048 SVs (49.6% precision) (Table 4). Combining the GATK and Long Ranger calls keeps recall roughly the same, but lowers the precision ∼10% (Supplemental Table 9). We also compared the LUMPY results to svclassify and found 1263 true positives (55.4% recall). Of note, the Long Ranger calls provide improved detection of larger SVs, with an expected bump around 300 bp, likely accounted for by better representation of *Alus* (Fig. 6).

Although sensitivity of the Long Ranger approach is good, this comes at the expense of specificity (Table 4; Supplemental Table 9). Given the bias in specificity in phased versus unphased regions, we expect that algorithmic improvements will produce further gains in sensitivity and specificity for this class of variants. In addition, the small number of events <200 bp in the svclassify set is likely not representative of the true number of calls but rather technical/algorithmic limitations.

### Analysis of samples from individuals with inherited disease

We went on to investigate the utility of Linked-Reads on samples with known disease-causing variants typically difficult to call with a standard, short-read exome. We obtained samples with known exon-level deletion and duplication events from a cohort that had been assessed using a high-depth NGS-based panel. We analyzed 12 samples from nine individuals using an Agilent SureSelect V6 Linked-Read exome at both 7.25 Gb (∼60-fold raw coverage) and 12 Gb (∼100-fold coverage) (Table 5). For three samples, patient-derived cell lines were available in addition to archival DNA, allowing investigation of the impact of DNA length on variant calling in this cohort.

**Table 5.**
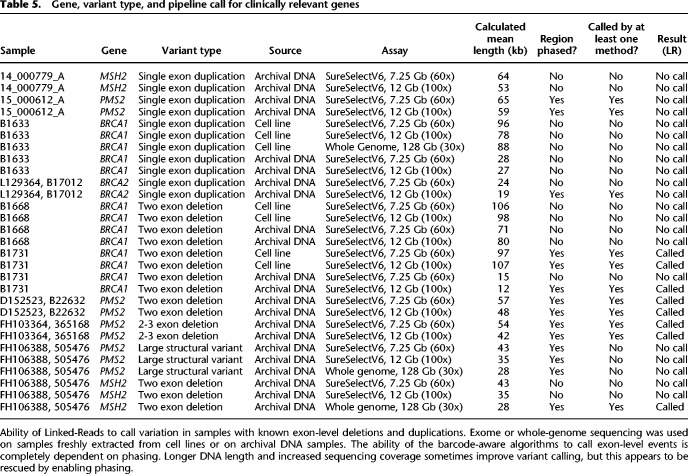
Gene, variant type, and pipeline call for clinically relevant genes

We identified five of the nine known exon-level events in these samples in at least one sample/depth combination. In two samples, increasing depth to 12 Gb enabled calling not possible at 7.25 Gb (Samples D and F [archival]) (Table 5). For the three samples with matched cell line and archival DNA, two had variants that could not be called in either sample type at either depth, whereas sample F could be called at both depths for the longer DNA extracted from the cell line, but could only be called at the higher depth in the shorter archival sample. Because the algorithms for calling these variants rely on phasing and barcode information, there is a correlation between gene phasing and variant calling, with no variants successfully called in samples not phased over the causative gene.

For two of the samples where Linked-Read exome sequencing was unable to phase or call the known variant, we performed lrWGS. In one case, the presence of intronic heterozygous variation was able to restore phasing to the gene and the known event was called. In the second case, there was insufficient heterozygous variation in the sample to allow phasing and the event was not called. This again demonstrates that phasing is dependent on molecule length as well as sample heterozygosity. In samples with decreased diversity in genes of interest, causative variant calling by Linked-Read sequencing was less likely (Supplemental Fig. 10). Generally, it should be possible to increase the probability of gene phasing in an exome assay by augmenting the bait set to provide coverage for common intronic variant SNPs. The addition of read coverage–based algorithms, such as those used with standard short-read exome sequencing, would also likely increase sensitivity in unphased regions, but were not used in this study.

One sample in this set contained both a single exon event and a large CNV in *PMS2*. Despite phasing of *PMS2* the variant was not called by genome or exome sequencing. Manual inspection of the data revealed increased phased barcode coverage in *PMS2*, supporting the presence of a large duplication missed by the SV calling algorithms, and providing evidence that improvements in the SV algorithms are possible (Supplemental Fig. 11).

## Discussion

Short-read sequencing has become the workhorse of human genomics. This cost effective, high-throughput, and accurate base calling approach provides robust analysis of short variants in unique regions of the genome, but struggles to reliably call SVs, cannot assess variation across the entire genome, and fails to reconstruct long range haplotypes (Sudmant et al. 2015). Recent studies have highlighted the importance of including haplotype information and more complete SV identification in genome studies (Chaisson et al. 2017). We have described an improved implementation of Linked-Reads coupled with novel algorithms in Long Ranger, that allows reconstruction of multi-megabase phase blocks, identification of large balanced and unbalanced SVs, and identification of small variants, even in regions of the genome typically recalcitrant to short-read approaches.

Some limitations to this approach currently exist. We observed a loss of coverage in regions of the genome with extreme GC content, and reduced performance in small indel calling, although this largely occurs in homopolymer regions and LCRs. Recent work suggests ambiguity in such regions may be tolerated for a large number of applications (Li et al. 2018). Although Linked-Reads can resolve many repetitive elements and genome regions, highly repetitive sequences that are larger than the length of input DNA are not resolvable by Linked-Reads. This limitation is common to all technologies currently available, including long-read sequencing. Repeat copies that reside on the same molecule will be subject to the same limitations as standard short-read approaches. It is clear that algorithmic improvements to Long Ranger would improve variant calling, particularly as some classes of variants, such as insertions, are not yet attempted. However, this is not uncommon for new data types, and there has already been some progress in this area (Elyanow et al. 2017; Spies et al. 2017; Karaoglanoglu et al. 2018; Xia et al. 2018). An additional limitation in this study is the reliance on a reference sample for calling variants, which creates reference bias and the inability to call variants in regions that are not resolved in the reference, as was the case with the SV in the pericentric region on Chromosome 16. To bypass any reference bias, Linked-Read data can also be used to perform diploid de novo assembly in combination with an assembly program, Supernova (Weisenfeld et al. 2017).

Despite these limitations, Linked-Read sequencing provides a clear advantage over short reads alone allowing for the construction of long range haplotypes as well as the identification of short variants and SVs from a single library and analysis pipeline. No other approach, to our knowledge, that scales to thousands of genomes provides this level of detail for genome analysis. Other recent studies have demonstrated the power of Linked-Reads to resolve complex variants in both germline and cancer samples (Collins et al. 2017; Greer et al. 2017; Nordlund et al. 2018; Viswanathan et al. 2018) and demonstrates that Linked-Reads outperforms the switch accuracy and phasing completeness of other haplotyping methods (Chaisson et al. 2017). The ability to represent and analyze genomes in terms of haplotypes, rather than compressed haploid representations, represents a crucial shift in our approach to genomics, allowing for a more complete and accurate reconstruction of individual genomes.

## Methods

### Samples and DNA isolation

Control samples (NA12878, NA19240, NA24385) were obtained as fresh cultured cells from the Coriell Cell Biorepository (https://catalog.coriell.org/1/NIGMS). DNA was isolated using the Qiagen MagAttract HMW DNA kit and quantified on a Qubit fluorometer following recommended protocols (https://support.10xgenomics.com/genome-exome/index/doc/user-guide-chromium-genome-reagent-kit-v2-chemistry).

Samples with known large SVs were obtained as cell lines from the NIGMS Human Genetic Cell Repository at the Coriell Institute for Medical Research (repository ID numbers listed in Supplemental Table 8). Frozen cell pellets were thawed rapidly at 37°C in 1 mL PBS. High molecular weight DNA was then extracted following recommended protocols, as above.

Clinical samples from individuals with known heterozygous variants in three Mendelian disease loci (*DYSF*, *POMT2*, and *TTN*) were collected at the Massachusetts General Hospital, Analytic and Translational Genetics Unit and shipped to 10x Genomics as cell lines and prepared as described above. Use of samples from the Broad Institute was approved by the Partners IRB (protocol 2013P001477).

Clinical samples from individuals with known exon-level del/dups were collected at The Institute of Cancer Research, London and shipped to 10x Genomics as cell lines or archival DNA. Samples were recruited through the Breast and Ovarian Cancer Susceptibility (BOCS) study and the Royal Marsden Hospital Cancer Series (RMHCS) study. All patients gave informed consent for use of their DNA in genetic research. The studies have been approved by the London Multicentre Research Ethics Committee (MREC/01/2/18) and Royal Marsden Research Ethics Committee (CCR1552), respectively. Samples were also obtained through clinical testing by the TGLclinical laboratory, an ISO 15189 accredited genetic testing laboratory. The consent given from patients tested through TGLclinical includes the option of consenting to the use of samples/data in research; all patients whose data was included in this study approved this option. DNA was extracted from cell lines as described above, and archival DNA samples were checked for size and quality according to the manufacturer's recommendations (https://support.10xgenomics.com/genome-exome/sample-prep/doc/demonstrated-protocol-hmw-dna-qc).

### Chromium Linked-Read library preparation

A Chromium controller chip was loaded with 1.25 ng of high molecular weight DNA, along with 10x Chromium reagents (either v1.0 or v2.0) and gel beads following recommended protocols (https://assets.contentful.com/an68im79xiti/4z5JA3C67KOyCE2ucacCM6/d05ce5fa3dc4282f3da5ae7296f2645b/CG00022_GenomeReagentKitUserGuide_RevC.pdf). Target enrichment for the Linked-Read whole-exome libraries was performed using Agilent SureSelect V6 exome baits following recommended protocols (https://assets.contentful.com/an68im79xiti/Zm2u8VlFa8qGYW4SGKG6e/4bddcc3cd60201388f7b82d241547086/CG000059_DemonstratedProtocolExome_RevC.pdf). Supplemental Figure 12 describes targeted sequencing with Linked-Reads.

### GemCode Linked-Read library preparation

For the GemCode comparator analyses, Linked-Read libraries were prepared for samples NA12878, NA12877, and NA12882 using a GemCode controller and GemCode V1 reagents following published protocols (Zheng et al. 2016).

### TruSeq PCR-free library preparation

Following recommended protocols (Supplemental Methods), 350–800 ng of genomic DNA was sheared to a size of ∼385 bp. Target enrichment for the Linked-Read whole-exome libraries was performed using Agilent SureSelect V6 exome baits following recommended protocols.

### Sequencing

Libraries were sequenced on a combination of Illumina instruments (HiSeq 2500, HiSeq 4000, and HiSeq X). Paired-End sequencing read lengths were as follows: TruSeq and Chromium whole-genome libraries (2×150 bp); Chromium whole-exome libraries (2×100 bp or 114 bp, 98 bp), and GemCode libraries (2×98 bp). lrWGS libraries are typically sequenced to 128 Gb, compared to 100 Gb for standard TruSeq PCR-free libraries. The additional sequence volume compensates for sequencing the barcodes as well as a small number of additional sources of wasted data and gives an average, deduplicated coverage of approximately 30×. To demonstrate the extra sequence volume is not the driver of the improved alignment coverage, we performed gene finishing comparisons at matched volume (100 Gb lrWGS and 100 Gb TruSeq PCR-) and continue to see coverage gains (Supplemental Fig. 12).

### Analysis

#### Comparison of 10x and GATK best practices

We ran the GATK best practices pipeline to generate variant calls for TruSeq PCR-free data using the latest GATK3.8 available at the time. We first subsample the reads to 30-fold whole-genome coverage. The read set is aligned to GRCh37 (hg19-2.2.0 reference using BWA-MEM version 0.7.12), reads are sorted, duplicates are marked, and the BAM is indexed using Picard tools (version 2.9.2; https://broadinstitute.github.io/picard/). Indel realignment and BAM recalibration (base quality score recalibration) is performed using known indels from Mills Gold Standard and The 1000 Genomes Project and variants from dbSNP (version 138). Indels and SNVs are called from the BAM using HaplotypeCaller and are genotyped to produce a single VCF file. For NA12878, this VCF file can be compared using hap.py (https://github.com/Illumina/hap.py, commit 6c907ce) to the truth variant set curated by Genome in a Bottle on confident regions of the genome. Sensitivity and specificity for both SNVs and indels is calculated to compare the Long Ranger short variant caller with the GATK Best Practices pipeline. All Long Ranger runs were performed with a prerelease build of Long Ranger version 2.2 utilizing GATK as a base variant caller. Long Ranger 2.2 has since been released.

#### Development of extended truth set

Any putative false positive variant found in the TruSeq/GATK or Chromium/Long Ranger VCFs was tested for support in the PacBio data (Supplemental Methods).

We selected regions of two- to sixfold degeneracy as determined by the “CRG Alignability” track (Derrien et al. 2012) as regions where improved alignment is likely to yield credible novel variants. We took the union of the GIAB confident regions BED file with these regions to determine the GIAB++ confident regions BED. The amount of sequence added to the GIAB++ BED differs by sample, as the original GIAB confident regions are sample-specific.

#### Structural variant comparison against deletion ground truth

After segmenting the Long Ranger deletion calls by size, we overlapped them to the svclassify set (Parikh et al. 2016) using the bedr package and BEDTools v2.27.1 (Quinlan and Hall 2010). We retained events >30 kb showing at least 50% reciprocal overlap. We also searched for Mendelian inheritance consistency on the parental samples NA12891 and NA12892. We annotated eight overlapping events with almost perfect breakpoint and Mendelian inheritance agreement. All genotypes were phased. In the svclassify overlapping deletions, all of the breakpoints except for the 3′ most in Chr 5: 104,432,114–104,503,672 had a read's length distance from each other. We then curated the remaining nine events called by Long Ranger that were not in the svclassify set. Of notice is one event (Chr 1: 189,704,517–189,783,347) contained within a larger deletion (Chr 1: 189,690,000–189,790,000). Among the nonoverlapping deletions, were six large SVs with breakpoint and Mendelian consistency in the parents. The other three (Chr 1: 189,690,000–189,790,000; Chr 11: 55,360,000–55,490,000; Chr 2: 242,900,000–243,080,000) had different breakpoints, were unphased but had consistent genotypes, or had no support in the parental data.

We took the Long Ranger deletion calls between 50 bp and 30 kb generated by both Long Ranger algorithms and GATK and merged them using SURVIVOR (Jeffares et al. 2017) allowing variants up to 50 bp apart to be merged. SURVIVOR was used again with a 50-bp merge distance to merge the Long Ranger deletion call set with deletions in the svclassify set. The resulting merged VCFs were then parsed to determine overlap and support for Long Ranger calls.

## Data access

All reference sample read data generated in this study have been submitted to the NCBI BioProject (BioProject; https://www.ncbi.nlm.nih.gov/bioproject/) under accession number PRJNA428496. Genomic short variation and structural variant study data for samples from individuals with inherited disease generated in this study have been submitted to the European Variation Archive (EVA; https://www.ebi.ac.uk/eva/) under accession number PRJEB28297. Genomic short variation and structural variant data for samples from the CNV Panel generated in this study have been submitted to the NCBI database of Genotypes and Phenotypes (dbGaP; https://www.ncbi.nlm.nih.gov/gap) under accession number phs001773.v1.p1. All code used in this paper is available online at GitHub. The lariat aligner code can be found at https://github.com/10XGenomics/lariat; the Long Ranger code is available at https://github.com/10XGenomics/longranger and as Supplemental Code; and specific analysis codes used in this paper can be accessed at https://github.com/10XGenomics/chromium-genome-paper.

## Competing interest statement

Patrick Marks, Sarah Garcia, Alvaro Martinez Barrio, Kamila Belhocine, Jorge Bernate, Rajiv Bharadwaj, Keith Bjornson, Claudia Catalanotti, Josh Delaney, Adrian Fehr, Ian Fiddes, Brendan Galvin, Haynes Heaton, Jill Herschleb, Christopher Hindson, Cassandra Jabara, Susanna Jett, Nikka Keivanfar, Sofia Kyriazopoulou-Panagiotopoulou, Bill Lin, Adam Lowe, Shamoni Maheshwari, Tony Makarewicz, Francesca Meschi, Christopher O'Keefe, Heather Ordonez, Pranav Patel, Andrew Price, Ariel Royall, Michael Schnall-Levin, Preyas Shah, David Stafford, Stephen Williams, Indira Wu, Andrew Wei Xu, and Deanna Church are current or former employees and stock holders of 10x Genomics.

## Supplementary Material

Supplemental Material
