# Supplementary material for: Resolving the full spectrum of human genome variation using Linked-Reads
Source: Genome Res. 2019 Apr;29(4):635–45. doi: 10.1101/gr.234443.118 (PMC6442396; doi:10.1101/gr.234443.118)
Supplement: Supplemental Material [file supp_29_4_635__index.html]

Resolving the full spectrum of human genome variation using Linked-Reads — Supplemental Material 

# Resolving the full spectrum of human genome variation using Linked-Reads

## Supplemental Material

- Supplemental\_Methods.pdf
- Supplemental\_FP\_validation.zip
- Supplemental\_longranger-2.2.zip
- Supplemental\_Mendelian\_inheritance\_analysis.zip
- Supplemental\_cov\_gain\_calculator.zip
- Supplemental\_Figures\_Tables.pdf
- Supplemental\_cov\_gain\_plots.zip
- Supplemental\_File\_S1.xlsx
- Supplemental\_evaluate\_dels\_svs.zip
- Supplemental\_FP\_read\_support\_counter.zip
