# Supplementary material for: Resolving the full spectrum of human genome variation using Linked-Reads
Source: Genome Res. 2019 Apr;29(4):635–45. doi: 10.1101/gr.234443.118 (PMC6442396; doi:10.1101/gr.234443.118)
Supplement: Supplemental Material [file supp_gr.234443.118_Supplemental_Methods.pdf]

Supplement to: Resolving the Full Spectrum of Human Genome Variation using Linked-Reads

Patrick Marks[a], Sarah Garcia[a], Alvaro Martinez Barrio[a], Kamila Belhocine[a], Jorge Bernate[a], Rajiv Bharadwaj[a], Keith Bjornson[a], Claudia Catalanotti[a], Josh Delaney[a], Adrian Fehr[a], Ian T. Fiddes [a], Brendan Galvin[a], Haynes Heaton[a,e,f], Jill Herschleb[a], Christopher Hindson[a], Esty Holt[b], Cassandra B. Jabara[a,g], Susanna Jett[a,h], Nikka Keivanfar[a], Sofia Kyriazopoulou-Panagiotopoulou[a,i], Monkol Lek[c,d], Bill Lin[a], Adam Lowe[a], Shazia Mahamdallie[b], Shamoni Maheshwari[a], Tony Makarewicz[a], Jamie Marshall[d], Francesca Meschi[a], Chris O'keefe[a], Heather Ordonez[a], Pranav Patel[a], Andrew Price[a], Ariel Royall[a], Elise Ruark[b], Sheila Seal[b], Michael Schnall-Levin[a], Preyas Shah[a], David Stafford[a], Stephen Williams[a], Indira Wu[a], Andrew Wei Xu[a], Nazneen Rahman[b], Daniel MacArthur[c,d], Deanna M. Church[a,j]

2019-01-24 23:07:26

Author affiliations a: 10x Genomics, 7068 Koll Center Parkway, Suite 401, Pleasanton, CA 94566; b: The Institute of Cancer Research, Division of Genetics & Epidemiology, 15 Cotswold Road, London, SM2 5NG, UK; c: Analytic and Translational Genetics Unit, Massachusetts General Hospital, Boston, MA; d: Program in Medical and Population Genetics, Broad Institute of MIT and Harvard, Cambridge, MA; e: Current affiliation, Wellcome Trust Sanger Institute, Hinxton CB10 1SA, UK; f: Current affiliation, University of Cambridge, Cambridge, UK; g: Current affiliation, Purigen Biosystems, Inc., 5700 Stoneridge Drive, Suite 100, Pleasanton, CA 94588; h: Current affiliation, LevitasBio, Inc., 3283 25th Street, 3, San Francisco, CA 94110; i: Current affiliation,

Illumina, Inc., 499 Illinois Street, Suite 201, San Francisco, CA 94158; j: Current affiliation, Inscripta Inc., 5500 Central Avenue #220, Boulder, CO 80301

Methods

1 Lariat aligner

Lariat is an aligner for barcoded Linked-Reads. All the Linked-Reads for a single barcode are aligned simultaneously, with the prior knowledge that the reads arise from a small number of long (10 kb - 200 kb) molecules. Lariat is an implementation of the RFA method (Bishara et al. 2015). Briefly, we model the observed reads for one barcode as being generated by a hierarchical process which first selects a small number of loci on the genome corresponding to the long input molecules, covering on average 500 kb of the genome. Then short reads are sampled with a uniform distribution over the selected loci. The sequencing process that generates the observed read sequence from the genome is modeled by the standard Smith-Waterman scoring scheme used by e.g. BWA-MEM (Li 2013). For each read a set of feasible candidate alignments is generated with traditional short-read alignment methods. An alignment configuration is a choice of one alignment from the feasible set for each read. We search for an alignment configuration that maximizes the likelihood of the data under the RFA model. A MAPQ can be derived from the likelihood ratio of the optimal alignment configuration to the sum of suboptimal configuration that select a different alignment for the read. The molecule selection process induces a strong prior that the aligned positions of reads cluster together on the genome. Reads with near-identical alignments to >1 locus would be assigned MAPQ < 10 in typical short-read data, and could not be used for variant calling. In the RFA method, confidently mapped reads flanking a duplicated region will anchor the molecule to the correct locus, and the molecular prior will strongly favor the alignments proximal to the confidently placed molecule, allowing the assignment of MAPQ>40 for reads with two identical alignments.

Lariat is written in the Go language and is available at https://github.com/10xGenomics/lariat. Upstream stages in the Long Ranger pipeline extract and correct the molecular barcodes, and prepare barcode sorted FASTQ-like inputs. Lariat generates candidate alignment positions by calling the BWA (Li 2013) API. It then performs RFA inference to select the final mapping position and MAPQ, and emits alignment records to BAM.

1.1 Adversarial alignments

We discovered a surprisingly high rate of degenerate alignments to segmental duplications with strong molecular evidence for one locus, but a better alignment score (typically by a single mismatch) at the other locus. In this case the RFA model will typically select the position supported by the molecular evidence, but with low MAPQ. Typically ~50% or 100% of reads mapped to the mismatch position supported the alternate allele suggesting the presence of a variant. We postulate that these cases are an expected feature of reference-based analysis of segmental duplications. Studies of copy-number in segmental duplication have cataloged singly unique nucleotides (SUNs), which are bases within one copy of a duplication that uniquely tag that copy (Sudmant et al. 2010). Typically SUNs alleles are introduced after the duplication event. Reads carrying the ancestral allele at a SUN position will be biased away from the SUN position to an ancestral copy. We term such reads 'adversarial' since the best alignment is not the correct mapping position.

We adopt a proposal from the RFA authors (A. Bishara, Y Lui, S. Batzoglou, private communication), to allow a collection of mapped reads from multiple barcodes to overturn reference alleles and realize a MAPQ improvement. Lariat implements a limited form of this approach. Reads are initially mapped in independent groups for each barcode. The second best alignment score and log-likelihood ratio of the molecular positioning analysis is stored in the BAM record. After mapping, Lariat scans the read pileups looking for sites with >= 3 alternate alleles among reads with molecule support for this location. In this case, the best explanation for the data is the presence of variant in the sample at this locus, rather than independent sequencing errors on

each read. We recompute the MAPQ for each read containing an alternate allele, but divide the mismatch error induced by the putative variant among all the reads supporting the variant. For isolated adversarial SNPs, MAPQ is typically increased from MAPQ=3 to MAPQ=40, leading to ~15,000 additional variant calls in degenerate regions.

1.2 Additional genome coverage gained with Linked-Reads and Lariat

To further investigate the properties of the parts of the genome with alignment coverage that is unique to a method, either Linked-Reads + Long Ranger (CrG) or PCR- short reads + BWA (TruSeq), we first looked at the distribution of these regions across the genome (Supplemental Figure S2). In the TruSeq data, the decoy sequence (hs37d5) has the greatest amount of unique sequence alignment in the two female samples, with the rest of the regions distributed roughly equally among the other chromosomes. This pattern is different for the one male sample (NA24385), where we see the largest sequence gain on the X chromosome.

In the CrG data, there is a completely different distribution of regions unique alignments. In all samples, chr9 shows the largest gain. This is driven by the ability to align sequences around the repetitive pericentromeric regions (Supplemental Figure S2). The ability of Lariat to resolve multi-mapping reads is a function of genome structure; the repeats need to be far enough apart that they are unlikely to share barcodes. This is reflected in the pattern of uniquely aligning sequence regions in CrG. Of note, there is a substantial gain of aligned regions on chrY in NA24385.

We then compared the uniquely aligning regions to exon and segmental duplication annotations. For both the TruSeq and CrG samples, we see roughly 5% of the uniquely aligning regions correspond to exon annotations using bedtools (Supplemental Table S1)(Quinlan and Hall 2010). For the TruSeq regions, we see a range of 17-30% of regions overlapping segmental duplications, and 13-28% aligning to the decoy. For the CrG samples, roughly 77% of the uniquely aligning regions correspond to segmental duplication annotations, and 12-14% to decoy alignments.

1.3 Comparison to PacBio

Raw PacBio FASTQs were aligned to the reference using BWA-MEM -x pacbio (Li 2013). To test a variant, we fetch all PacBio reads covering the variant position, and retain the substring aligned within 50 bp of the variant on the reference. We re-align the PacBio read sequence to the +/-50 bp interval of the reference, and the same interval with the alternate allele applied. A read is considered to support the alternate allele if the alignment score to the alt-edited template exceeds the alignment score of the reference template. A variant was considered to be validated if at least 2 PacBio reads supported the alt allele, at least 10 PacBio reads covered the locus, and the overall alternate allele fraction seen in the PacBio reads was at least 25%.

2 Variant Phasing

A variety of methods for phasing haplotypes have been proposed, which optimize a variety of different objective functions (Bansal et al. 2008; Bansal and Bafna 2008). The HASH (Bansal et al. 2008) phasing method optimizes the likelihood of generative probabilistic objective function that is a natural model of reads generated from haplotypes. The method was designed for ~800 bp Sanger reads as input fragments. HASH uses a MCMC approach to optimize the objective function, which may lead to long running times.

We build on this basic approach, and extend the probabilistic model to be robust to mixed fragments which contain alleles from both haplotypes. We add a new variant hypothesis we term ‘non-heterozygous’ that allows the model to identify variants that were initially called as heterozygous but whose alleles do not cleanly segregate onto the local haplotpes, which may be false-positive calls, or homozygous variants incorrectly called as heterozygous. We use local realignment to both allele sequences to carefully quantify the allele supported by a read, which is critical for good phasing performance of indel variants. Finally we develop an efficient search heuristic that combines direct beam-search to phase chunks of ~50 variants, a greedy stitching pass

to phase chunks to each other, and final polishing pass to correct any local errors.

2.1 Introduction

We take as input a pre-determined set of of biallelic variants. We label the alleles $A_{i,p}$ where $i \in 1, ..., N$ indexes the variant, and $p \in 0, 1$ is an arbitrary label for the two alleles of the variant.

The set of alleles that come from the same parent chromosome is referred to as a haplotype, and are arbitrarily labeled H_0 and H_1. The goal of the phasing algorithm is to determine which allele from each variant came from each parent chromosome. The phasing result can be described by a binary variable for each variant $X_i \in 0, 1$ where $X_i = 0$ indicates the $A_{i,0} \in H_0$ and $A_{i,1} \in H_1$ and $X_i = 1$ indicates that $A_{i,0} \in H_1$ and $A_{i,1} \in H_0$.

Neighboring variants on the genome are often separated by distances longer than the read-pair length, causing very short phase blocks. Long input fragments covering a small fraction (0.001) of the genome are exposed to each barcode, so the probability that a barcode contains reads from both haplotypes is small.

We cast the solution to the phasing problem as a search for the maximum likelihood phasing parity vector:

$$\hat{\mathbf{X}} = \underset{\mathbf{X}}{\operatorname{argmax}} P(\mathbf{O}|\mathbf{X})$$

where $\mathbf{O}$ denotes the sets of barcoded reads observed, and $\mathbf{X}$ is the phasing result we wish to infer.

Read pairs are aligned to the genome as usual. Reads are grouped by the attached barcode sequences. Reads with common barcodes are partitioned into groups that are likely to have originated from a single genomic input fragment, and thus provide evidence that the alleles covered by the reads came from the same haplotype.

We compute the probability of the observed reads covering variant i from fragment f as:

$$\log P(O_{i,f}|A_{i,p}) = \sum_{r \in O_{i,f}} \mathbf{1}(S_r = A_{i,p})(1 - 10^{-Q_r/10}) + \mathbf{1}(S_r \neq A_{i,p})(10^{-Q_r/10})$$

where r sums over reads, $\mathbf{1}(S_r = A_{i,p})$ is the indicator function testing if the rth sequence S_r match allele $A_{i,p}$. The probability assigned is derived from the inverse-Phred transformed quality value of relevant read base Q_r.

The data from a fragment f comes from one of three cases. First two cases are that the alleles present are only from H_0 or only from H_1. These cases are the typical case and have a high prior probability, governed by the fraction of the genome present in each partition. The third case is that input DNA from both haplotypes was present at the locus, so both either allele is equally likely to be observed:

$$P(O_{1,f}, ...O_{N,f}|\mathbf{X}, H_f = 0) = \prod_i P(O_{i,f}|A_{i,X_i})$$

$$P(O_{1,f}, ...O_{N,f}|\mathbf{X}, H_f = 1) = \prod_i P(O_{i,f}|A_{i,1-X_i})$$

$$P(O_{1,f}, ...O_{N,f}|\mathbf{X}, H_f = M) = \prod_i 0.5$$

These equations give the probability of the observed reads from fragment f at variant location i, X_i, and fragment haplotype H_f. Observations are independent given the variant party and fragment haplotype. The prior probability of third case is α – the probability that a partition contains both haplotypes at a locus. We can then compute the overall likelihood by summing over the three cases:

$$P(O_{1,f}, ...O_{N,f}|\mathbf{X}) = \frac{(1-\alpha)}{2}(\prod_i P(O_{i,f}|A_{i,X_i}) + \prod_i P(O_{i,f}|A_{i,1-X_i})) + \alpha \prod_i 0.5$$

Fragments are independent given the variant party X_i letting us form the overall objective

function as:

$$P(\mathbf{O}|\mathbf{X}) = \prod_f P(O_{1,f}, ...O_{N,f}|\mathbf{X})$$

2.2 Optimization

We optimize the overall objective function using a hierarchical search over the phasing vector $\mathbf{X}$.

Initially we break up $\mathbf{X}$ into local chunks of $n \approx 40$ variants and determine the relative phasing of the block using beam search over the assignments of $X_k, X_{k+1}, ...X_{k+n}$. Where k is the first variant in the local block. Beam search is a standard method that has existed for a long time (see http://en.wikipedia.org/wiki/Beam_search).

The relative phasing of neighboring blocks is found greedily, yielding a candidate phasing vector $\mathbf{X}$. Finally $\mathbf{X}$ is iteratively refined by swapping the phase of individual variants. When refinement converges we are left with our estimate of the optimal phasing configuration $\hat{\mathbf{X}}$.

2.3 QV Testing

We can compute estimates of the accuracy of the phasing configuration by computing the likelihood ratio between the optimal configuration $\hat{\mathbf{X}}$ and some alternate configuration $\mathbf{X}_{alt}$ by computing the likelihood ratio between the hypotheses. The confidence is then reported as a Phred-scaled quality value:

$$Q(\mathbf{X}_{alt}) = -10\log_{10}(\frac{P(\mathbf{O}|\mathbf{X}_{alt})}{P(\mathbf{O}|\hat{\mathbf{X}}})$$

There are two classes of errors we consider: short switch errors and long switch errors. Short switch errors are single variants that are assigned the wrong phasing in an otherwise correctly phased region - to measure the short switch confidence of variant i, we flip X_i to form $\mathbf{X}_{alt}$. When the short switch confidence is low, the variant is marked as not phased in the output, rather than reporting a phasing call likely to be erroneous.

Long switch errors occur when two neighboring blocks of variants $..., X_{i-2}, X_{i-1}$ and $X_i, X_{i+1}, ...$ are correctly phased internally, but have the wrong relative phasing between the two blocks. In this case we say a long switch error occurred at position i. We test the long switch confidence at position i by inverting the phase of X_j for all $i >= j$. When the long switch confidence falls below a threshold we start a new phase block – variants in different phase blocks are not called as phased with respect to one another.

3 SV calling from linked-reads

3.1 Finding candidate regions with a lot of barcode overlap

The goal of this step is to get a high-sensitivity/low-specificity list of potential SV candidates. Given two loci, we want a quick way to decide whether they share a significant number of common barcodes. The list of these loci will go into the next step of the algorithm, which uses a probabilistic calculation to make a more accurate prediction as to whether the observed barcode overlap is consistent with the presence of a structural variant.

Expected barcode overlap between distant loci If the two loci are on different chromosomes or the distance between them is much larger than the average molecule length, then we can use a binomial test to determine if the observed barcode overlap between the loci is larger than expected by chance. Let N_1, N_2, and N be the observed number of barcodes at the first locus, the observed number of barcodes at the second locus, and the barcode diversity respectively. Then, the probability of observing n common barcodes between the two loci is governed by the binomial distribution:

$$Binom(n; N_1, N_2/N)$$

Therefore, we can pick a p-value cutoff and select all pairs of loci for which the above probability is less than our cutoff. These loci will serve as candidates for distal SVs.

Expected barcode overlap between not so distant loci The binomial test above assumes that

the two loci under consideration are independent in that no molecule can span both loci. This assumption clearly does not hold when the distance d between two loci is in the order of the molecule length.

Given the count of barcodes on each of the loci and the distance between them, we want to compute the expected number of common barcodes between the two loci. We start by computing the probability that a molecule with barcode b present at locus X will reach locus $X + d$.

$$f_b(d) = P(b \text{ present at } X + d | b \text{ present at } X) =$$

$$sum_{m:L(m)>d}(P(\text{molecule at } X \text{ is } m)P(m \text{ present at } X + d | m \text{ present at } X)$$

Here the sum is over molecules m from barcode b with length $L(m) > d$. The first probability above is $L(m)/sum_{m'}L(m')$. The second is $(L(m) - d)/L(m)$. So after simplifying we get $sum_{m:L(m)>d}(L(m) - d))/sum_{m'}(L(m'))$. In practice, we get good results when simplifying this to $sum_{m:L(m)>d}L(m)/sum_{m'}L(m')$.

Given two loci at distance d with N_1 and N_2 barcodes respectively, we estimate the expected barcode overlap between them as

$$min(N_1, N_2) \times f(d)$$

where $f(d) = \text{avg}_b f_b(d)$

In practice, we pre-compute $f(d)$ for a range of values of d. We can also further reduce the time required to compute $f(d)$ by sampling a large number of barcodes instead of using all of them to compute the above average.

3.2 Probabilistic model

3.2.1 Setting up the maximum-likelihood problem

Given two candidate loci for structural variation, we want to determine whether the observed reads in the two loci are more consistent with the presence or the absence of an SV. In particular, we want to find the model that maximizes the data (log-)likelihood

$$\log P(D; m) = \sum_b \log P(D_d; m)$$

Here, D_b is the observed data from barcode b (at the loci of interest - the presence of the barcode at very distant loci is considered irrelevant). Data from different barcodes are independent (conditioning on the model). m is the model and comes from a discrete set of models:

1. no SV (no-SV or reference model),
2. homozygous SV at loci x and y, or
3. SV at loci x and y on haplotypes i and j respectively.

Here, x and y could be any pair of loci of the genome, but in practice we only consider a relatively small list of loci pairs, based for example on barcode overlaps or read-pair support. i and j are in $\{0, 1\}$ and denote the haplotype assignment of the breakpoints x and y. We further assume that if x and y are on the same phase block, then i and j must be equal (i.e. the SV-calling cannot redefine phase blocks). We can further refine this set of SV models based on the type of the SV (more on this later).

There are two sets of latent variables, $H_b^{x,y}$ the haplotype assignment of barcode b at loci x and y, and M_b, the number of molecules from which the reads with barcode b were generated. For simplicity, we assume that M_b can be at most 2, since it is extremely unlikely that there are more than two molecules from the same locus in the same partition (or that we had multiple partitions with the same barcode).

Below is summary of notation:

1. D is the observed data (positions of reads, their barcodes, and the ph) in the loci under consideration.
2. D_b is the data (i.e. read positions) from barcode b.
3. $D_{b_{1\ldots k}}$ is a subset of D_b comprising the first k reads from barcode b.
4. R_b is the event that there is no SV on barcode b (or that b was generated from the reference).
5. $SV_b^{x,y}$ is the event that there is an SV between positions x and y on the haplotype that generated b.
6. $SV_{ij}^{x,y}$ is the event that there is an SV at positions x, y on haplotypes i and j respectively, where $i, j \in \{0, 1\}$.
7. We assume that reads are generated from a Poisson distribution with rate α (uniform across the genome). That is, α is the expected number of reads per basepair.
8. $P_L(\ell)$ is the probability of having a molecule of length ℓ. In practice, we use the empirical molecule length distribution.
9. $L_{\max}$ is the maximum possible length of an input molecule.

3.2.2 Some useful probabilities

Probability of a molecule Let $x_{b_1} \leq x_{b_2} \leq \ldots \leq x_{b_n}$ be the positions of the reads from a single molecule with barcode b. We assume that the reads are generated from a single molecule with hidden length ℓ. The distances $x_{b_{i+1}} - x_{b_i}$ are the waiting times between events of a Poisson process. The log-probability of observing the molecule is:

$$
\begin{aligned}
&\log P_m(x_{b_1}, x_{b_2}, \ldots, x_{b_n}) = \\
&\log \left[\sum_{\ell \geq x_{b_n} - x_{b_1}} P_L(\ell) \alpha e^{-\alpha(\ell - (x_{b_n} - x_{b_1}))} \prod_{i=1}^{n-1} \alpha e^{-\alpha(x_{b_{i+1}} - x_{b_i})} \right] = \\
&\log \left[\alpha^n \prod_{i=1}^{n-1} e^{-\alpha(x_{b_{i+1}} - x_{b_i})} \sum_{\ell \geq x_{b_n} - x_{b_1}} P_L(\ell) e^{-\alpha(\ell - (x_{b_n} - x_{b_1}))} \right] = \\
&\log \left[\alpha^n e^{-\alpha(x_{b_n} - x_{b_1})} \sum_{\ell \geq x_{b_n} - x_{b_1}} P_L(\ell) e^{-\alpha(\ell - (x_{b_n} - x_{b_1}))} \right] = \\
&\log \left[\alpha^n \sum_{\ell \geq x_{b_n} - x_{b_1}} P_L(\ell) e^{-\alpha \ell} \right] = \\
&n \log \alpha + \text{logaddexp}_{\ell \geq x_{b_n} - x_{b_1}} \left[\log P_L(\ell) - \alpha \ell \right]
\end{aligned}
$$

where logaddexp is the log of the sum of the exponentials of the arguments. Intuitively, the probability of observing the molecule is the product of the following probabilities:

1. The probability of getting a molecule of length ℓ given that the molecule length was greater than $x_{b_n} - x_{b_1}$.
2. The probability of observing waiting times $x_{b_{i+1}} - x_{b_i}$.
3. The probability of observing no reads in a length $\ell - (x_{b_n} - x_{b_1})$.

These probabilities are then summed over all possible lengths $\ell \geq x_{b_n} - x_{b_1}$. Since P_m only depends on the observed length $d = x_{b_n} - x_{b_1}$ and the number of reads n, below we will also use the (overloaded) notation $P_m(n, d)$.

Barcode likelihood assuming no SV The likelihood of the data from barcode b assuming that all of the data from barcode b were generated from a single molecule from the reference is:

$$P(D_b | M_b = 1; R_b) = P_m(n, d)$$

if $x_{b_1}, \ldots, x_{b_n}$ are all on the same chromosome and $x_{b_n} - x_{b_1} < L_{\max}$. Otherwise,

$P(D_b|M_b = 1; R_b) = \epsilon$.

Similarly:

$$P(D_b|M_b = 2; R_b) = \sum_{k=2}^{n-1} P(D_{b_{1\ldots k}}|M_{b_{1\ldots k}} = 1; R_{b_{1\ldots k}})P(D_{b_{k+1\ldots n}}|M_{b_{k+1\ldots n}} = 1; R_{b_{k+1\ldots n}})$$

More accurately, we would sum over all possible splits into two disjoint subsets. However, this would add too much complexity (especially given how unlikely barcode collisions are and how few molecules are typically within a partition), so we assume that molecules cannot overlap but can "touch".

Barcode likelihood assuming a homozygous SV The likelihood assuming that the data from barcode b were generated from an SV haplotype $P(D_b|M_b = 1; SV_b^{x,y})$ depends on the type of the SV.

Deletions Assume that the SV is a deletion between x and y ($x < y$) and that $x_{b_i} < x \leq x_{b_{i+1}}$ and $x_{b_j} < y \leq x_{b_{j+1}}$.

1. If $x > x_{b_n}$ or $y < x_{b_1}$ then $P(D_b|M_b = 1, SV_b^{x,y}) = P(D_b|M_b = 1; R_b)$. We assume that SVs are independent from each other, in that we can only have at most one SV within the length of a molecule.
2. If $i \neq j$, this means that the molecule has reads inside the deletion, so $P(D_b|M_b = 1, SV_b^{x,y}) = \epsilon$.
3. If none of the above holds, we have $x_{b_1} \leq x_{b_2} \leq \ldots \leq x_{b_i} < x < y \leq x_{b_{i+1}} \leq \ldots \leq x_{b_n}$. Let $d = y - x$ be the length of the deleted sequence. Then $P(D_b|M_b = 1, SV_b^{x,y}) = P_m(x_{b_1}, x_{b_2}, \ldots, x_{b_i}, x_{b_{i+1}} - d, \ldots, x_{b_n} - d) = P_m(n, x_{b_n} - x_{b_1} - d)$.

To compute $P(D_b|M_b = 2, SV_b^{x,y})$ we again need to consider all splits of the reads from barcode b into two chunks. Like before we simplify by only considering non-overlapping chunks.

$$P(D_b|M_b = 2; SV_b^{x,y}) = \sum_{k=2}^{n-1} P(D_{b_{1\ldots k}}|M_b = 1; SV_b^{x,y}) P(D_{b_{k+1\ldots n}}|M_b = 1; SV_b^{x,y})$$

Depending on where x_k is with respect to x and y each of the probabilities above is equal to the probability under either the reference or the SV model.

Inversions Assume that the SV is an inversion between x and y ($x < y$) and that $x_{b_i} < x \leq x_{b_{i+1}}$ and $x_{b_j} < y \leq x_{b_{j+1}}$.

1. If $x_{b_1} \leq x_{b_2} \leq \ldots \leq x_{b_i} < x \leq x_{b_{i+1}} \leq \ldots \leq x_{b_n} < y$ (reads span x but end before y) or $x < x_{b_1} \leq \ldots x_{b_i} < y \leq \ldots \leq x_{b_n}$ (reads start after x and span y). In the first case, $P(D_b|M_b = 1, SV_b^{x,y}) = P_m(x_{b_1}, x_{b_2}, \ldots, x_{b_i}, d - x_{b_n}, d - x_{b_{n-1}}, \ldots, d - x_{b_{i+1}}) = P_m(n, x - x_{b_1} + y - x_{b_{i+1}}) = P_m(n, d - x_{b_1} - x_{b_{i+1}})$, where $d = x + y$. The second case is similar.
2. In all other cases (reads entirely before x, reads entirely after y, reads entirely between x and y, or reads spanning across x and y), $P(D_b|M_b = 1, SV_b^{x,y}) = P(D_b|M_b = 1; R_b)$.

Duplications Assume that the SV is a duplication between x and y ($x < y$) and that $x_{b_i} < x \leq x_{b_{i+1}}$ and $x_{b_j} < y \leq x_{b_{j+1}}$.

1. If $x < x_{b_1}$ and $y > x_{b_n}$, then the reads span the duplication and $P(D_b|M_b = 1, SV_b^{x,y}) = P_m(n, d + y - x)$.
2. If $x < x_{b_1}$ and $y > x_{b_n}$ (reads entirely within the duplication), then $P(D_b|M_b = 1, SV_b^{x,y}) = \max\Big(P_m(n, x_{b_n} - x_{b_1}), \max_j P_m(n, y - x - x_{b_{j+1}} + x_{b_j})\Big)$.
3. Otherwise, $P(D_b|M_b = 1, SV_b^{x,y}) = P(D_b|M_b = 1; R_b)$.

Large-scale translocations We only consider this case if $x_{b_1}, \ldots, x_{b_n}$ are generated from two different chromosomes or $x_{b_n} - x_{b_1} > L_{\max}$. We can then split the reads into two groups $x'_{b_1}, \ldots, x_{b'_n}, x''_{b_1}, \ldots, x''_{b''_n}$ such that $n' + n'' = n$. Each group contains the subset of reads closer to

x and y respectively.

1. If any of the two sets of reads above are empty then $P(D_b|M_b = 1, SV_b^{x,y}) = P(D_b|M_b = 1; R_b)$.
2. If $x'_{b'_n} < x$ and $x''_{b''_1} > y$, then $P(D_b|M_b = 1, SV_b^{x,y}) = P_m(n, x - x'_{b_1} + x''_{b''_n} - y)$. All cases where all reads from the first set are on the same side of x and all reads from the second set are on the same side of y are similar.
3. Otherwise, $P(D_b|M_b = 1, SV_b^{x,y}) = \epsilon$.

Unknowns Since Long Ranger identifies event types by matching to simple models of deletions, duplications and inversions, there are additional events where Long Ranger identifies clear evidence for anomalous barcode overlap or coverage, but is unable to match the event to one of the pre-defined models, these are labeled "unknown".

3.3 EM

We can use an EM approach to maximize the likelihood. This involves repeatedly conditioning on the latent variables to compute the maximum likelihood model and then getting a posterior estimate of the latent variables.

3.3.1 M-step: Likelihood conditioning on the latent variables

Homozygous reference The likelihood of the data under the homozygous reference model is:

$$\prod_d \sum_{c=1}^{2} P(D_b|M_b = c, R_b)P(M_b = c)$$

Homozygous SV The likelihood of the data under the homozygous SV model is:

$$\prod_d \sum_{c=1}^{2} P(D_d|M_b = c; SV_b^{x,y})P(M_b = c)$$

We need to compute this for every type of SV.

Heterozygous SV

$$P(D_b;m) = \sum_{i,j\in[0,1]^2} \sum_{c=1}^{2} P(D_b|H_b^{x,y}=(i,j),M_b=c;m)P(H_b^{x,y}=(i,j),M_b=c;m)$$

where m is the model (reference or SV).

$$P(D_b|H_b^{x,y}=(i,j),M_b=1;SV_{i,j}^{x,y}) = P(D_b|SV_b^{x,y},M_b=1)$$

$$P(D_b|H_b^{x,y}\neq(i,j),M_b=1;SV_{i,j}^{x,y}) = P(D_b|R_b,M_b=1)$$

To compute $P(D_b|H_b^{x,y}=(i,j),M_b=2;SV_{i,j}^{x,y})$, we start with the case where x and y are on the same phase block, so i and j must be equal.

$$P(D_b|H_b^{x,y}=(i,i),M_b=2;SV_{i,i}^{x,y}) =$$
$$\sum_{k=2}^{n-1} P(D_{b_{1\ldots k}}|H_{b_{1\ldots k}}^{x,y}=(i,i),M_b=1;SV_{i,i}^{x,y})P(D_{b_{k+1\ldots n}}|H_{b_{1\ldots k}}^{x,y}=(i,i),M_b=1;SV_{i,i}^{x,y})$$

Here the sum is taken over all ways of splitting the reads from $b, x_1, x_2, \ldots, x_n$ into two (non empty) sequences $x_1,\ldots,x_k$ and $x_{k+1},\ldots,x_n$. $D_{b_{1\ldots k}}$ and $D_{b_{k+1\ldots n}}$ are the sets of reads resulting from such a split. Depending where x_k is with respect to x $P(D_{b_{1\ldots k}}|H_{b_{1\ldots k}}^{x,y}=(i,i),M_b=1;SV_{i,i}^{x,y})$ is either $P(D_{b_{1\ldots k}}|R_{b_{1\ldots k}},M_{b_{1\ldots k}}=1)$ or $P(D_{b_{1\ldots k}}|SV_{b_{1\ldots k}}^{x,y},M_{b_{1\ldots k}}=1)$. The sum is taken similarly for the likelihood of the second chunk of data.

If x and y are on different phase blocks, then i and j can be different. To simplify things a bit, we assume that the only valid split is the one that assigns the points closer to x to haplotype i and the

points closer to y to haplotype j. The computation is then similar to the case above.

3.3.2 E-step: Posterior of the latent variables

$$P(H_b^{x,y} = (i,j), M_b = c | D_b; m) \propto P(D_b | H_b^{x,y} = (i,j), M_b = c; m) P(H_b^{x,y} = (i,j), M_b = c)$$

All we need is a prior on the latent variables. First, we assume that

$$P(H_b^{x,y} = (i,j), M_b = c) = P(H_b^{x,y} = (i,j)) P(M_b = c)$$

To compute $P(H_b^{x,y} = (i,j))$, let $p_b^x(0), p_b^x(1)$ be the probability that barcode b at locus x is phased on haplotype 0 or 1 respectively. We assume that these probabilities are pre-computed during SNP phasing. If b is un-phased at x, then we can set $p_b^x(0)$ to 0.5 or to the fraction of barcodes that are phased to haplotype 0 at locus x. If x and y are in the same phase set, then $P(H_b^{x,y} = (i,j)) = p_b^x(i)$ if $i == j$ and $P(H_b^{x,y} = (i,j)) = 0$ otherwise. If x and y are on different phase blocks, then $P(H_b^{x,y} = (i,j)) = p_b^x(i) p_b^y(j)$.

To compute $P(M_b = c)$, let p_{ov} be the probability of having two overlapping molecules in the same partition. The probability that the reads with barcode b came from a single molecule is the product of the probability of generating a molecule greater than the observed length and the probability that there is no molecule overlap: $P(M_b = 1) = \sum_{\ell \geq x_{b_n} - x_{b_1}} P_L(\ell)(1 - p_{ov})$ and $P(M_b = 2) = 1 - P(M_b = 1)$.

3.4 Computing SV phasing scores

We can assign a score to the haplotype assignment of the SV as follows:

$$\frac{P(D; SV^{x,y}_{i,j})}{\sum_{(i,j)\in[0,1]^2} P(D; SV^{x,y}_{i,j})}$$

3.5 Barcode coverage

The genomic extent of long input molecules is inferred by 'linking' successive read-pairs with the same barcode if there are separated by <60 kb. A barcode coverage track is computed by counting the number of inferred molecules that span each position in the genome.

4 SV calling details

4.1 Large SVs (>30 kb)

Inheritency consistency analysis for NA12878

To evaluate 1) calls in the svclassify set that were not called by Long Ranger and 2) PASS SV calls contained in NA12878 that were not in the svclassify set, we looked to Long Ranger analyses of the parental samples for evidence of these events as well as manual review of the barcode overlap and coverage data.

With regard to svclassify events not called by Long Ranger- one event (chr12:8,558,486-8,590,846) is well-supported in the Linked-Read data by barcode overlap. For this event, Long Ranger calls a 10 kb small deletion with a consistent 5' breakpoint to the svclassify event, but prematurely closes the event, missing 22 kb of the deletion. The event is called correctly in both parents. A second event (chr22:24,274,143-24,311,297) is also well-supported in all three individuals and is called by Long Ranger but is filtered out from PASS and moved to Filtered as it overlaps with a segmental duplication and is thus an error of annotation. There is no support for the last missing call (chr14:37,631,608-37,771,227). Further investigation of this call reveals that it is genotyped as homozygous reference in NA12878 in the 1000G SV set (Sudmant et al. 2015), and represents an

error in the svclassify set relative to GRCh37.p13.

The three Long Ranger-only events that did not show inheritance consistency with breakpoint consistency all overlap more complex events/regions of the genome. The first, chr1:189690000-189790000, entirely contains an event that does show inheritance and breakpoint consistency (chr1:189704517-189783347). The second, chr11:55360000-55490000, overlaps with an event annotated as UNK (chr11:55365428-55445878) that is inheritance and breakpoint consistent with NA12891 and thus represents a more complex event than just a simple deletion. The final event, chr2:242900000-243080000, overlaps with four known GRCh37 assembly issues (HG-1616, HG-1709, HG-1714, and HG-1911), and is immediately upstream of a known assembly gap. We see a drop in phased coverage on haplotype 2 in NA12878 that is not seen in either parent, and thus this likely represents a false positive call in a complex assembly region (Supplemental Table S7).

Details on GeT-RM CNVPanel samples To assess for performance of Linked-Reads on clinically-relevant variant types typically assessed by aCGH or karyotype we performed 30× Linked-Read genome sequencing on a set of 23 samples with known balanced or unbalanced SVs. These 23 samples are derived from 1) the GeT-Rm CNVPanel, a collection of unbalanced events including large deletions, duplications, inversions, balanced translocations and unbalanced translocations designed to assess performance of clinical aCGH or 2) the Coriell general Cell Repository (balanced events). These cell lines have multiple, orthogonal assays confirming the presence of their described structural variants. We assessed the performance of each of the barcode-based detection methods (barcode coverage and barcode overlap) individually in our panel of samples covering a wide range of structural variant types (Supplemental Table S1). Together, the barcode overlap and barcode coverage methods detected 27 of the 29 structural variants, correctly characterizing 22 of the 23 samples tested. The barcode coverage method called all copy number variants except one; a chr7 deletion filtered to a secondary list of candidate structural variants because the breakpoints overlap a segmental duplication, a feature known to complicate genome analysis. However, this event was detected with the barcode overlap method, demonstrating the advantage of utilizing multiple detection methods for variant calling. The barcode overlap method

is unable to detect terminal events because it requires the ability to examine barcode sharing patterns on both sides of a breakpoint. This algorithm was able to call all non-terminal copy number events and all balanced events, except one. This undetected event is a balanced translocation with a breakpoint in a heterochromatic region of chromosome 16. This region is represented by Ns in the reference assembly and will be invisible to any sequence-based method relying on the reference genome (Schneider et al. 2017).

Downsampling of sequence data was performed in silico to determine the minimum sequence depth required to detect each CNV. Due to the evenness provided by barcode coverage, the deletion and duplication signals for these two samples are detectable even with coverage as low as 5 Gb (~1× genomic read coverage) (Supplemental Figure S8 C and D). The barcode overlap method was less robust with reduced sequencing and did not call CNVs with less than 50 Gb of sequence. This result is expected, given that the algorithm was designed for use with full-depth data. However, there was an observable signal in the barcode overlap data with as little as 5-10 Gb for many of the samples, indicating that the algorithm is likely extensible to lower depth data. (Supplemental Figure S9).

5 TruSeq PCR-free library preparation

350-800 ng of genomic DNA was sheared to a size of ~385 bp using a CovarisM220 Focused Ultrasonicator using the following shearing parameters: Duty factor = 20%, cycles per burst = 200, time = 90 seconds, Peak power 50. Fragmented DNA was then cleaned up with 0.8× SPRI beads and left bound to the beads. Then, using the KAPA Library Preparation Kit reagents (KAPA Biosystems, Catalog # KK8223), DNA fragments bound to the SPRI beads were subjected to end repair, A-base tailing and Illumina‘PCR-free’ TruSeq adapter ligation (1.5 M final concentration of adapter was used). Following adapter ligation, two consecutive SPRI cleanup steps (1.0× and 0.7×) were performed to remove adapter dimers and library fragments below ~150 bp in size. No library PCR amplification enrichment was performed. Libraries were then eluted off the SPRI beads in 25

μl elution buffer and quantified with quantitative PCR using KAPA Library Quant kit (KAPA Biosystems, Catalog # KK4824) and an Agilent Bioanalyzer High Sensitivity Chip (Agilent Technologies) following the manufacturer's recommendations.

References

Bansal V, Bafna V. 2008. HapCUT: An efficient and accurate algorithm for the haplotype assembly problem. *Bioinformatics* **24**: i153–i159.

Bansal V, Halpern AL, Axelrod N, Bafna V. 2008. An MCMC algorithm for haplotype assembly from whole-genome sequence data. *Genome Res* **18**: 1336–1346.

Bishara A, Liu Y, Weng Z, Kashef-Haghighi D, Newburger DE, West R, Sidow A, Batzoglou S. 2015. Read clouds uncover variation in complex regions of the human genome. *Genome Res* **25**: 1570–1580.

Li H. 2013. Aligning sequence reads , clone sequences and assembly contigs with BWA-MEM. *ArXiv* **00**: 1–2.

Quinlan AR, Hall IM. 2010. BEDTools: A flexible suite of utilities for comparing genomic features. *Bioinformatics* **26**: 841–842.

Schneider VA, Graves-Lindsay T, Howe K, Bouk N, Chen H-C, Kitts PA, Murphy TD, Pruitt KD, Thibaud-Nissen F, Albracht D, et al. 2017. Evaluation of grch38 and de novo haploid genome assemblies demonstrates the enduring quality of the reference assembly. *Genome Res* **27**: 849–864.

Sudmant PH, Kitzman JO, Antonacci F, Alkan C, Malig M, Tsalenko A, Sampas N, Bruhn L, Shendure J, 1000 Genomes Project, et al. 2010. Diversity of human copy number variation and multicopy genes. *Science* **330**: 641–646.
