# Supplementary material for: Resolving the full spectrum of human genome variation using Linked-Reads
Source: Genome Res. 2019 Apr;29(4):635–45. doi: 10.1101/gr.234443.118 (PMC6442396; doi:10.1101/gr.234443.118)
Supplement: Supplemental Material [file supp_gr.234443.118_Supplemental_Figures_Tables.pdf]

Supplementary Figures and Tables for: Resolving the Full Spectrum of Human Genome Variation using Linked-Reads

Patrick Marks[a], Sarah Garcia[a], Alvaro Martinez Barrio[a], Kamila Belhocine[a], Jorge Bernate[a], Rajiv Bharadwaj[a], Keith Bjornson[a], Claudia Catalanotti[a], Josh Delaney[a], Adrian Fehr[a], Ian T. Fiddes [a], Brendan Galvin[a], Haynes Heaton[a,e,f], Jill Herschleb[a], Christopher Hindson[a], Esty Holt[b], Cassandra B. Jabara[a,g], Susanna Jett[a,h], Nikka Keivanfar[a], Sofia Kyriazopoulou-Panagiotopoulou[a,i], Monkol Lek[c,d], Bill Lin[a], Adam Lowe[a], Shazia Mahamdallie[b], Shamoni Maheshwari[a], Tony Makarewicz[a], Jamie Marshall[d], Francesca Meschi[a], Chris O'keefe[a], Heather Ordonez[a], Pranav Patel[a], Andrew Price[a], Ariel Royall[a], Elise Ruark[b], Sheila Seal[b], Michael Schnall-Levin[a], Preyas Shah[a], David Stafford[a], Stephen Williams[a], Indira Wu[a], Andrew Wei Xu[a], Nazneen Rahman[b], Daniel MacArthur[c,d], Deanna M. Church[a,j]

2019-02-01 16:52:56

Author affiliations a: 10x Genomics, 7068 Koll Center Parkway, Suite 401, Pleasanton, CA 94566; b: The Institute of Cancer Research, Division of Genetics & Epidemiology, 15 Cotswold Road, London, SM2 5NG, UK; c: Analytic and Translational Genetics Unit, Massachusetts General Hospital, Boston, MA; d: Program in Medical and Population Genetics, Broad Institute of MIT and Harvard, Cambridge, MA; e: Current affiliation, Wellcome Trust Sanger Institute, Hinxton CB10

1SA, UK; f: Current affiliation, University of Cambridge, Cambridge, UK; g: Current affiliation, Purigen Biosystems, Inc., 5700 Stoneridge Drive, Suite 100, Pleasanton, CA 94588; h: Current affiliation, LevitasBio, Inc., 3283 25th Street, 3, San Francisco, CA 94110; i: Current affiliation, Illumina, Inc., 499 Illinois Street, Suite 201, San Francisco, CA 94158; j: Current affiliation, Inscripta Inc., 5500 Central Avenue #220, Boulder, CO 80301

Supplemental Figures

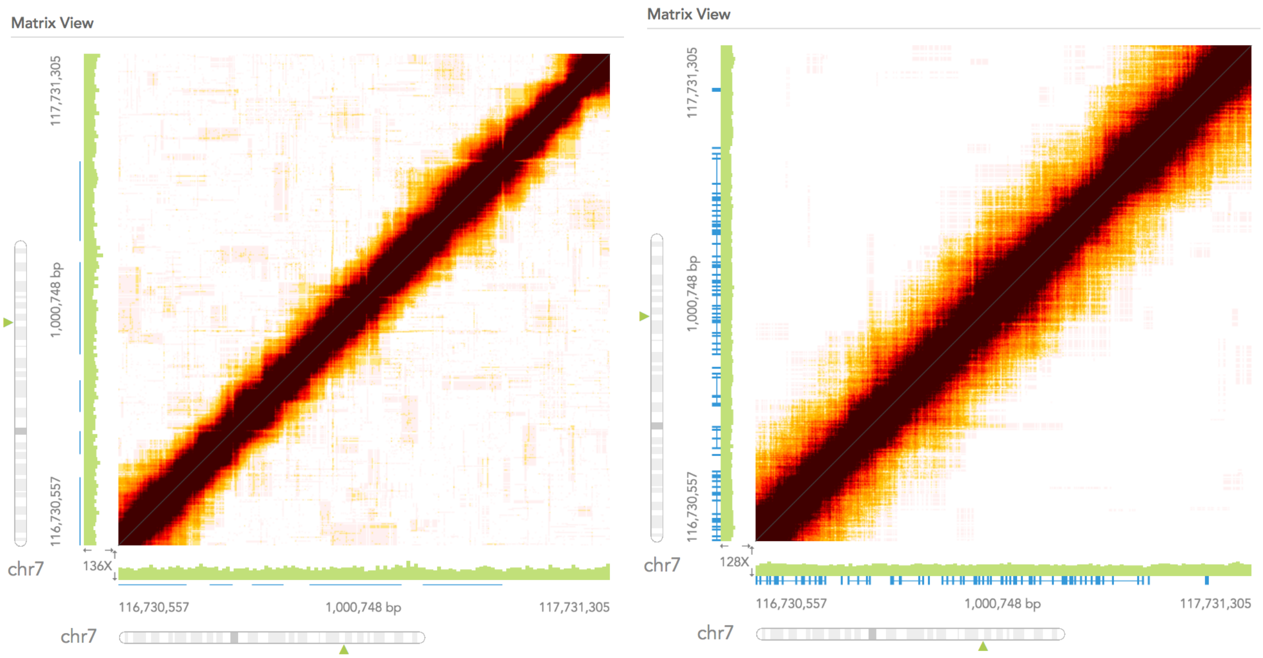

Figure 1: Improvements in Chromium Genome relative to GemCode. Loupe browser screenshots of GemCode data (left) and Chromium Genome data (right) showing barcode overlap patterns plotted between a region on chromosome 7 and itself. Overlap is strongest along the diagonal, with decreasing overlap occurring as a function of distance. Off of the diagonal there is more barcode sharing in the GemCode data (indicated by the light orange background signal) due to increased barcode collisions owing to the reduced number of barcodes and partitions in the GemCode assay.

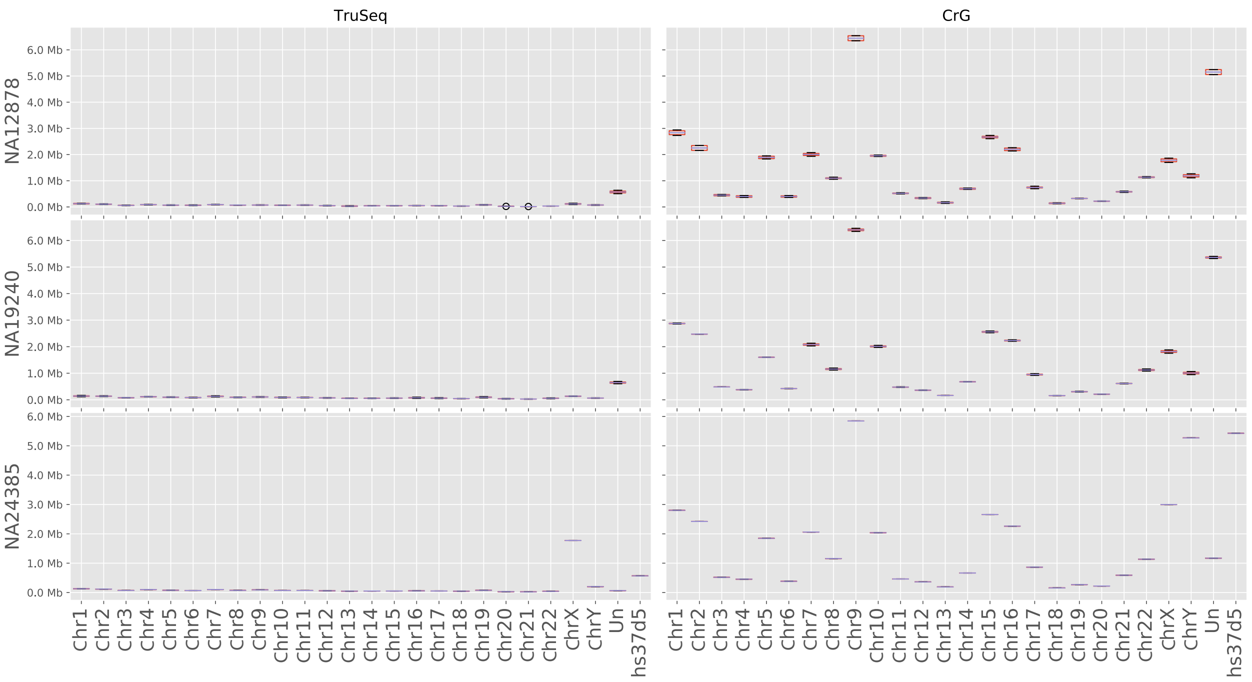

Figure 2: Uniquely aligning sequence by chromosome. Data in the left column is from PCR- TruSeq, and in the right column is lrWGS. The y-axis represents the amount of sequence covered by the unique alignments. The x-axis represents the chromosome assignment of the regions being assessed. The top row represents 4 replicates from the NA12878 sample, second row represents 2 replicates from the NA19240 sample, and the third row represents 1 replicate of NA24385.

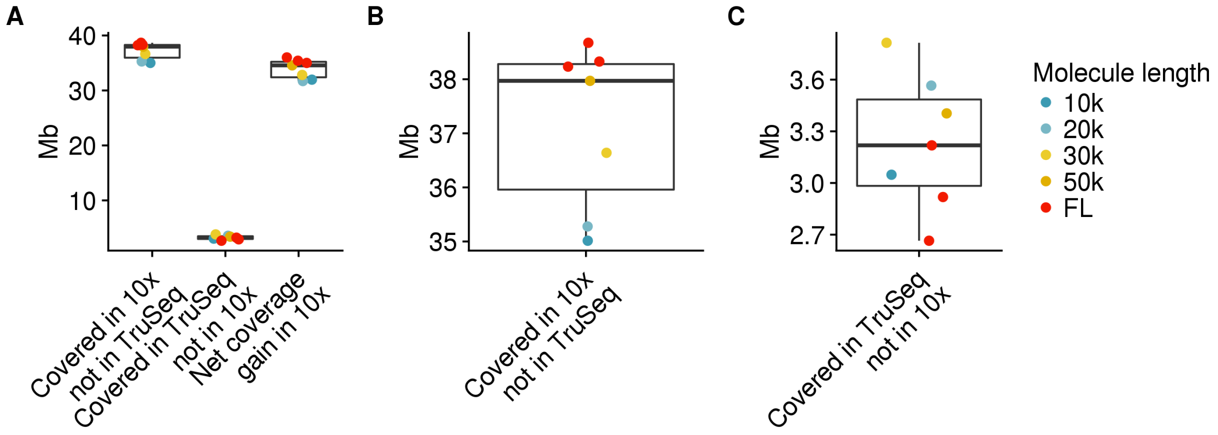

Figure 3: Increasing input molecule length improves 10x Chromium Genome alignments. The y-axis shows the amount of sequence with a coverage of >=5 reads at mapQ30 in one genome assay but not the other. All comparisons use data from the NA12878 cell line. Panel (B) and (C) are zoomed-in views of box plots in (A).

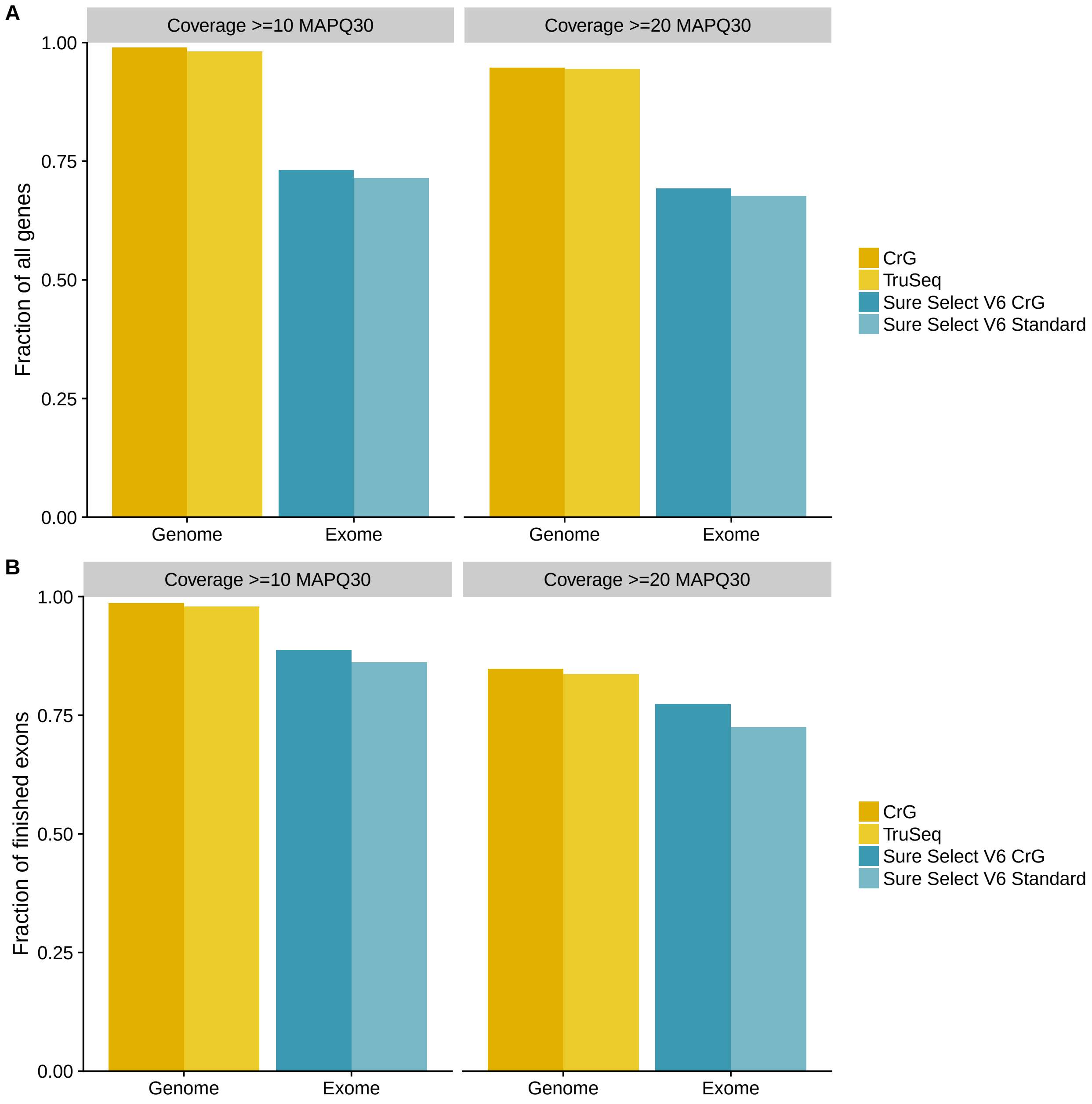

Figure 4: Improved coverage and gene finishing across the genome and exome. Upper panel shows fraction of all genes for which coverage is greater than 10 or 20 reads, with MapQ30 or greater. Lower panel shows fraction of finished exons for which more than 99 percent of bases within an exon meet the same coverage and quality metrics. In each panel, Genome is shown in yellow, Exome in blue.

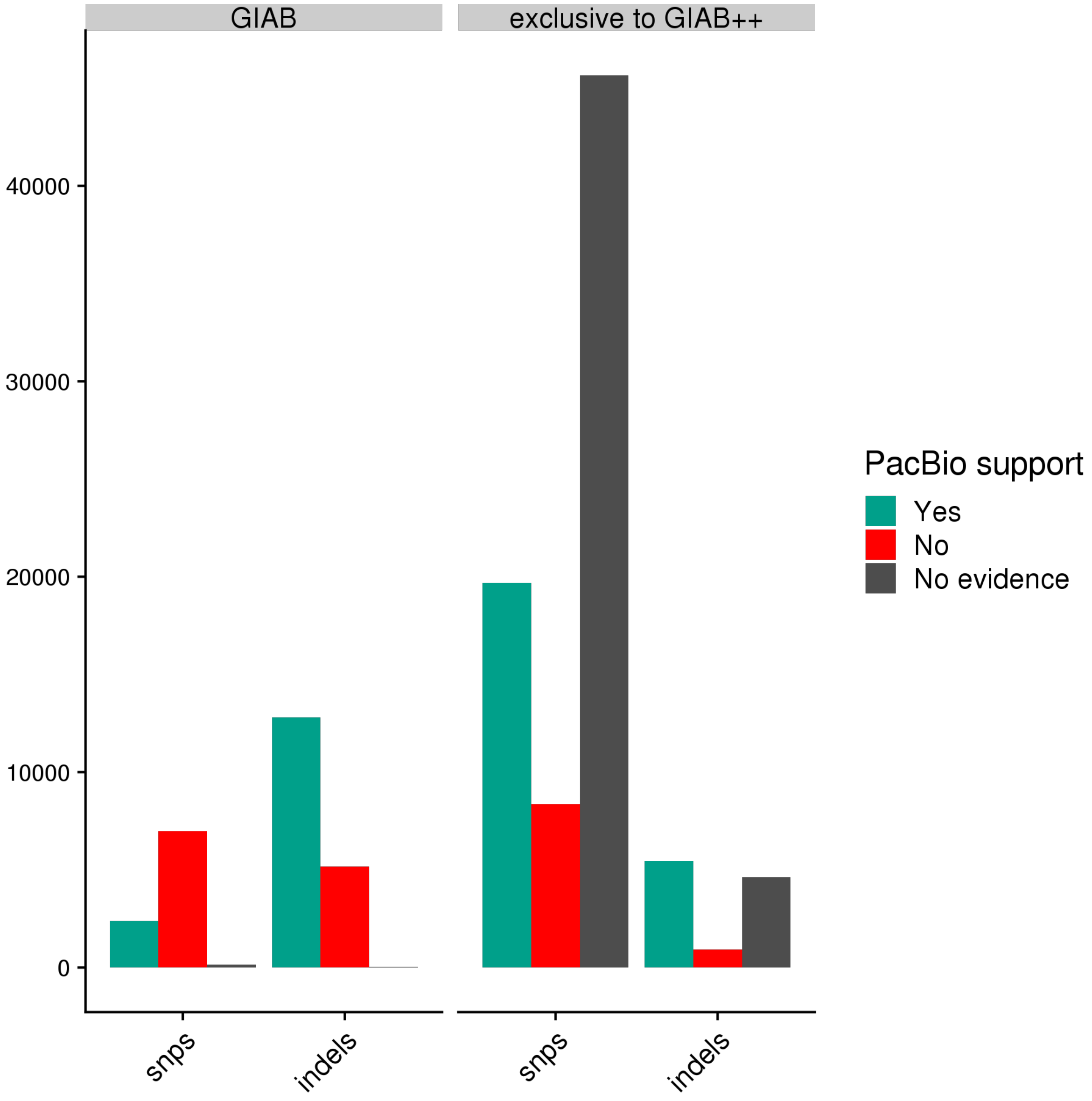

Figure 5: Many putative False Positive calls have PacBio support. On left is the number of putative false positive calls for which PacBio shows support (green), does not show support (red), or lacks coverage over the region (<10reads, grey). On right is the same analysis across the extended truth set.

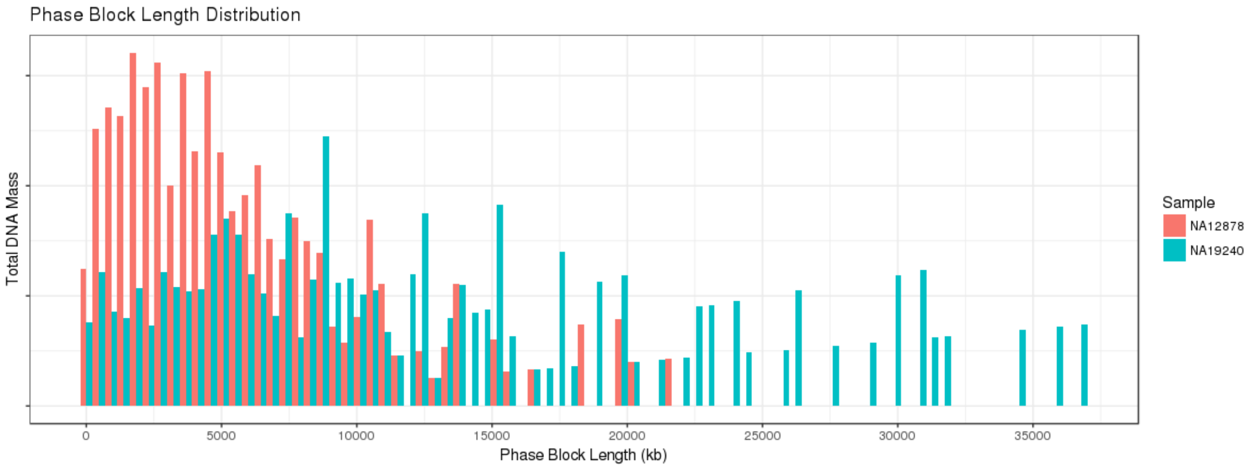

Figure 6: Phase block distribution of NA19240 and NA12878. Length weighted phase block length distribution of input molecule length matched NA12878 and NA19240. Both samples have an input molecule length around 80 kb, but the phase block length distribution is larger for NA19240 due to the increased heterozygosity in this sample. Phase block lengths for both samples were taken from the phase_blocks.h5 file generated by Long Ranger and plotted as the length weighted histogram of phase block lengths.

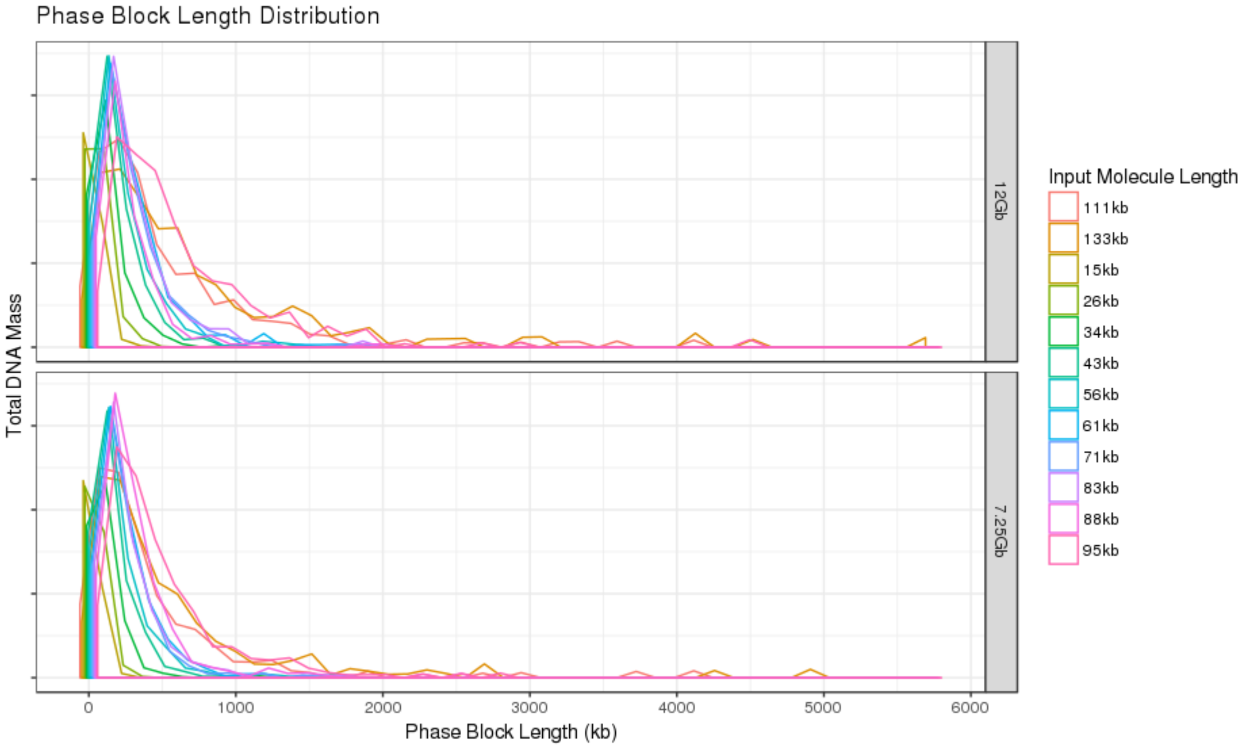

Figure 7: Impact of molecule length on phase block distributions of exome samples from individuals with inherited disease. Length weighted phase-block size distribution for clinical exome samples at 7.25 Gb and 12 Gb sequencing depth, colored by input molecule length. Longer input molecule length leads to longer phase blocks. Phase block lengths for both samples were taken from the phase_blocks.h5 file generated by Long Ranger and plotted as the length weighted histogram of phase block lengths.

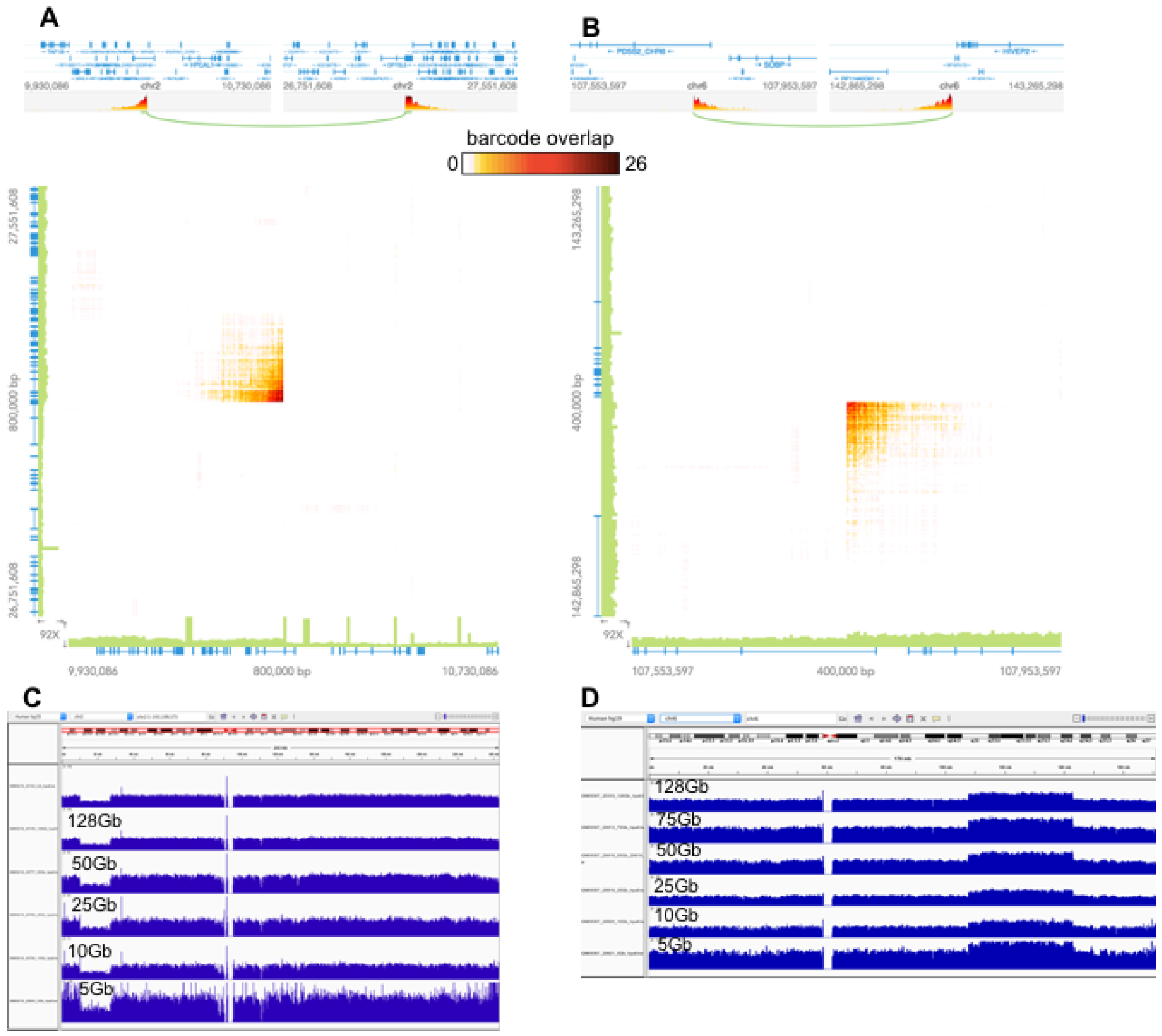

Figure 8: Copy number variants detected with barcode overlap and barcode coverage. Visualizations of a deletion event in sample GM09261: 46,XY,del(2)(p25.1p23) (A and C) and a duplication event in sample GM09367: 46,XX,dup(6)(q21q24) (B and D). A, B. Barcode overlap linear (top) and matrix (bottom) views of these events with 128 Gb sequence. These events were not called by barcode overlap at lower sequence depths. C, D. IGV tracks showing barcode coverage in the event regions with sequence depths of 128 Gb down to 5 Gb, as indicated. Both events were called by the barcode coverage method at all sequence depths tested.

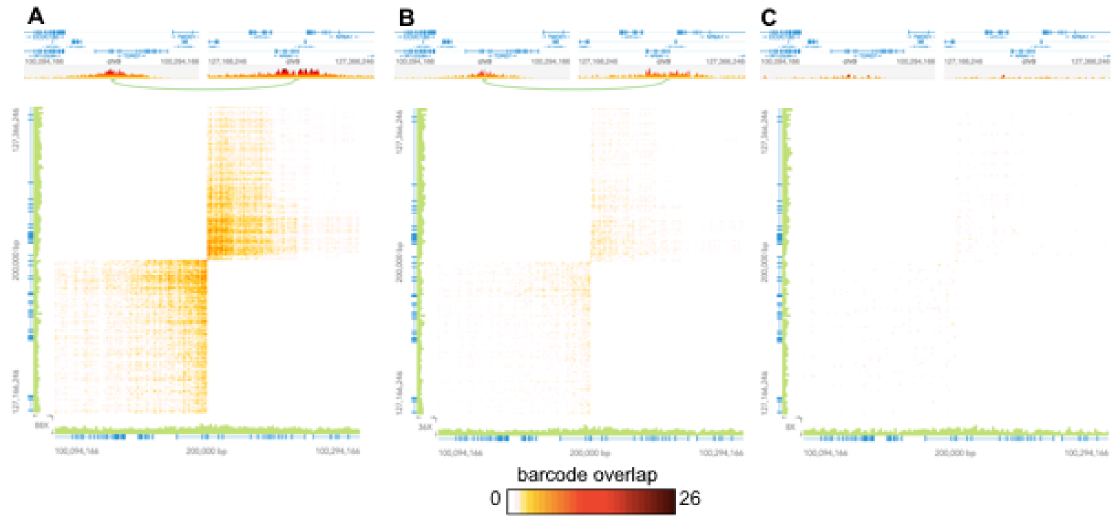

Figure 9: Detection of event for GM21075: 46,XY,inv(9)(q22.3q34.1). A. Barcode Matrix view showing a balanced inversion detected on the long arm of chromosome 9 with 128 Gb of Linked-Read sequence data. B. Barcode matrix view of the same inversion event shown with only 50 Gb of Linked-Read sequence coverage, the lowest coverage at which Long Ranger called this event. C. The same event shown with 10 Gb of Linked-Read sequence coverage showing that there is signal for this event in the data at this coverage level, even though Long Ranger does not make the definitive call.

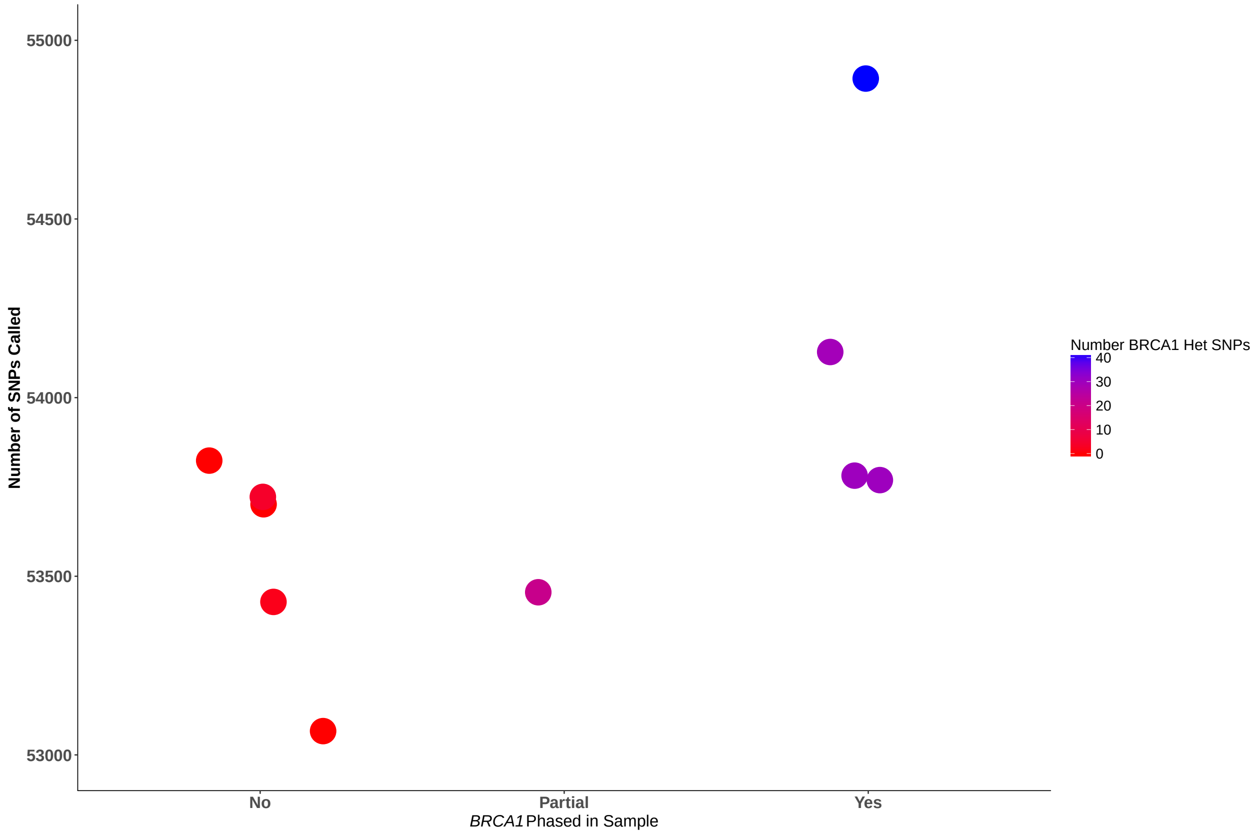

Figure 10: Comparison of variant density at the *BRCA1* locus in samples where *BRCA1* is phased vs. when it is not phased. Samples with less variation fail to phase.

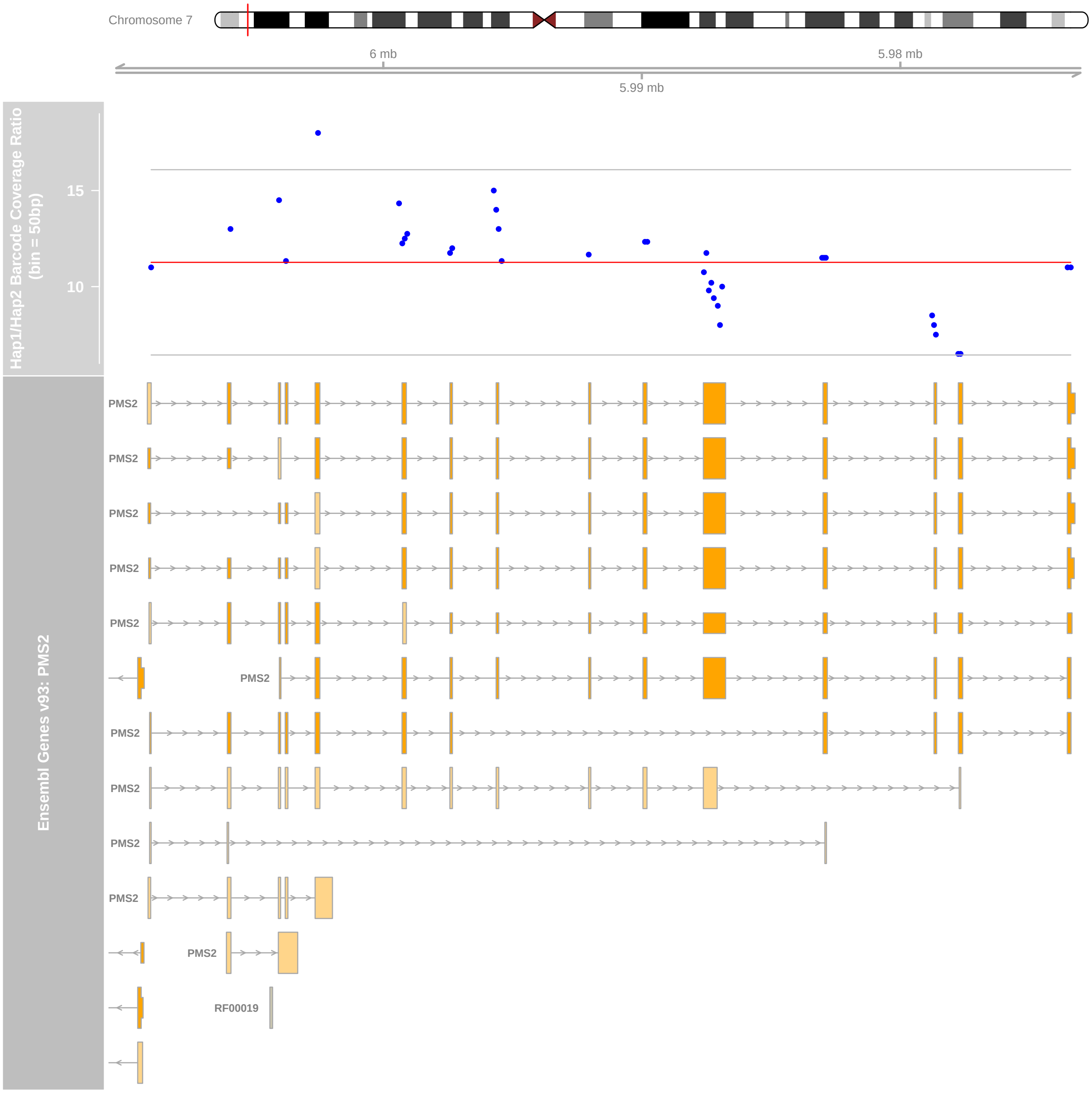

Figure 11: 50 bp binned exome barcode coverage over the *PMS2* region in sample I showing evidence for duplication. Dashed grey lines indicate mean +/- 2 standard deviations and red line is the mean. Below is shown the *PMS2* Ensembl v93 gene track.

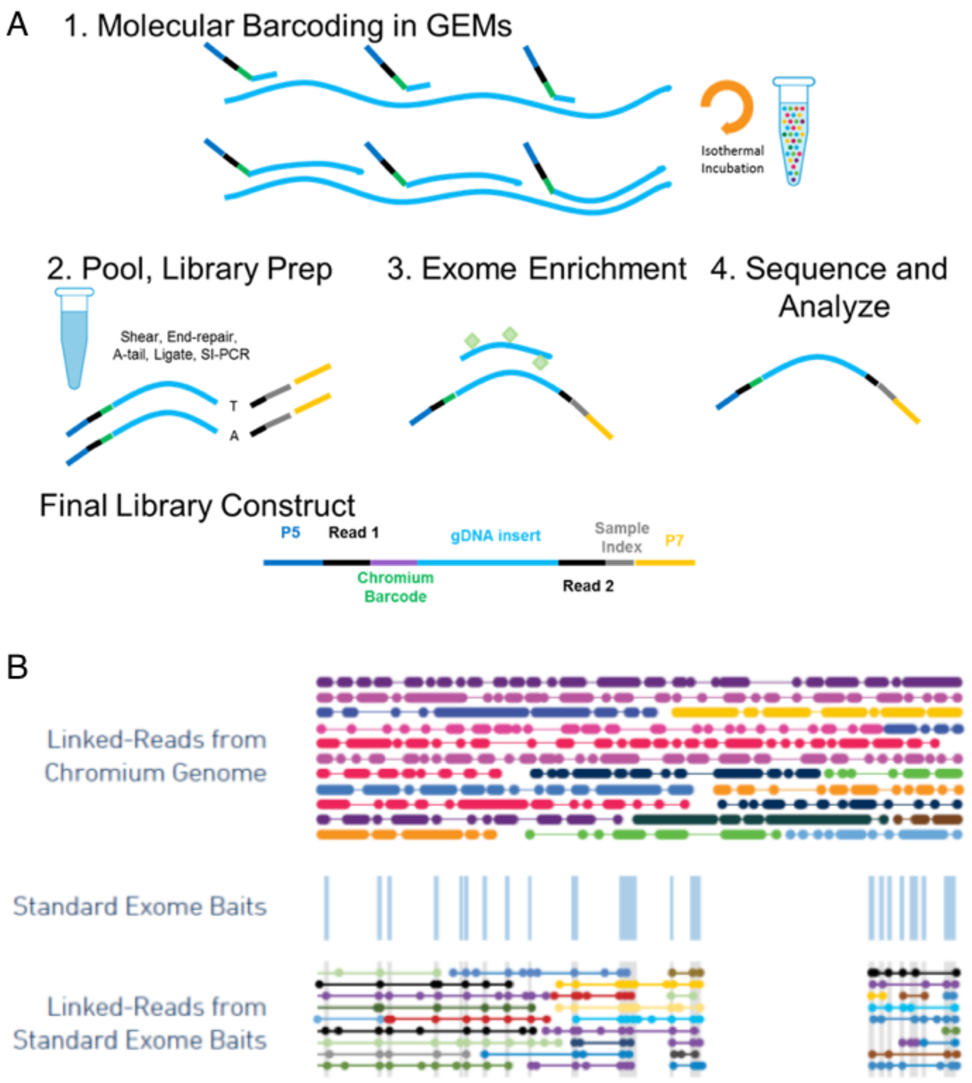

Figure 12: Exome capture scheme. A. The Chromium Exome workflow includes industry-standard library preparation steps, exome capture, and standard short-read sequencing. Barcoded gel beads are mixed with high-molecular weight DNA and enzyme mixture, then combined with an oil-surfactant solution in a double-cross microfluidic junction. Gel bead droplets are collected and dissolved, and whole-genome primer extension is initiated to generate barcoded fragments. Barcoded fragments are pooled and can be used for final library preparation. B. Exome baits can be used to isolate genic content for exome sequencing. Barcoded fragments are assembled into Linked-Reads using barcode identity and physical proximity by alignment. Linked-Reads are amenable to standard capture for maintenance of long-range phasing and/or performance over regions of interest.

Table 1: Comparison of uniquely aligning sequence per assay to genome annotation information

Sample	Sex	Method	Unique aligning seq	Unique in exon	% in exon	Unique in SD	% in SD	Unique in decoy	% in decoy
NA12878	F	CrG	36454253	1838221	5.04%	28301860	77.64%	5054699	13.87%
NA24385	M	CrG	44231881	2124519	4.80%	33896875	76.63%	5425578	12.27%
NA19240	F	CrG	37643806	1849618	4.91%	28699783	76.24%	5401123	14.35%
NA12878	F	Tru	2151952	109503	5.09%	635160	29.52%	591793	27.50%
NA24385	M	Tru	4122860	169393	4.11%	700745	17.00%	568695	13.79%
NA19240	F	Tru	2991088	175180	5.86%	690759	23.09%	690858	23.10%

Table 1: Comparison of uniquely aligning sequence per assay to genome annotation information. We took 1 replicate from each sample and compared the uniquely aligning sequence to regions annotated as segmental duplication (SD), regions annotated as exonic per Ensembl annotation, or the human decoy sequence (hs37d5). For each comparison, both absolute number of bases and percentage of bases are provided.

Table 2: Sensitivity and Specificity

Variable	CrG NA12878	PCR- NA12878	CrG NA24385	PCR- NA24385
Sensitivity (het SNVs)	0.995	0.997	0.996	0.998
Specificity (het SNVs)	0.996	0.998	0.997	0.999
Sensitivity (het indels)	0.952	0.984	0.953	0.988
Specificity (het indels)	0.956	0.987	0.955	0.990
Sensitivity (homalt SNVs)	0.999	0.999	0.999	0.999
Specificity (homalt SNVs)	0.999	1.000	0.999	1.000
Sensitivity (homalt indels)	0.988	0.996	0.986	0.997
Specificity (homalt indels)	0.941	0.974	0.939	0.975
Sensitivity (het SNVs) (++)	0.992	0.994	0.995	0.997
Specificity (het SNVs) (++)	0.970	0.984	0.966	0.980
Sensitivity (het indels) (++)	0.940	0.974	0.926	0.983
Specificity (het indels) (++)	0.926	0.966	0.886	0.939
Sensitivity (homalt SNVs) (++)	0.998	0.997	0.999	0.998
Specificity (homalt SNVs) (++)	0.982	0.995	0.977	0.991
Sensitivity (homalt indels) (++)	0.981	0.991	0.955	0.993
Specificity (homalt indels) (++)	0.918	0.959	0.882	0.922

Table 2: Sensitivity/specificity by inferred zygosity. Hap.py was used to determine the error rate of variants called by Long Ranger for NA12878 and NA24385 in both the GIAB confident regions as well as the ++ confident regions. The extended summary results are tabulated here, reporting on the sensitivity and specificity of heterozygous and homozygous variant calls.

Table 3: Manual review of small variant calling

Ex	Location	Manual assessment	Quality	LR genotype	GIAB genotype	Supporting reads/ Total reads Hap1	Supporting reads/ Total reads Hap2	Supporting reads/ Total reads PB	Supporting reads/ Total reads TruSeq
1	chr1: 569427:C:T	TP	1205.77	0/1	./.	15/15	25/25	7/52	13/14
2	chr2:21106587:T:C	TP	578.77	0/1	./.	10/10	20/20	1/25	13/29
3	chr2:120171025:G:A	TP	164.77	0/1	./.	0/20	15/16	0/41	22/53
4	chr1:91227215:G:A	FP	673.77	0/1	1/1	0	0	42/43	36/36
5	chr1:174312140:G:A	FP	190.77	0/1	./.	0/12	0/5	2/49	1/36
6	chr10:31964340:G:C	TP	606.77	0/1	./.	0/14	9/9	0/60	9/33
7	chr10:120532871:T:A	FP	127.77	0/1	./.	0/0	0/0	2/50	0/22
8	chr13:69502758:G:T	FP	391.77	0/1	./.	0/0	0/0	1/55	0/21
9	chr14:59356314:C:A	TP	678.78	1|1	0/1	0/1	3/3	34/52	21/21
10	chr15:72150190:T:C	FP	67.77	0/1	./.	4/7	0/17	0/48	6/38
11	chr17:1132584:C:T	TP	415.77	1/1	0/1	0/6	4/4	19/47	10/24
12	chr18:4101856:G:A	FP	85.77	0/1	./.	4/24	1/14	0/49	1/23
13	chr18:41688063:G:A	TP	733.77	0/1	./.	11/11	0/16	3/48	13/25
14	chr19:16496584:T:A	FP	60	0/1	./.	0/7	0/6	0/63	4/31
15	chr2:10614364:G:A	TP	161.77	0/1	./.	0/13	16/16	5/34	16/30
16	chr2:120171060:A:C	TP	198.77	0/1	./.	0/25	16/16	1/42	24/59
17	chr2:153864925:A:G	TP	101.77	0/1	./.	0/9	2/2	13/33	0/4
18	chr2:242916020:T:C	TP	450.77	0/1	1/1	9/9	0/13	15/45	8/21
19	chr3:8963428:C:T	FP	76.77	0/1	./.	0/0	0/0	0/38	1/25
20	chr3:119160680:G:A	TP	429.77	0/1	./.	8/8	0/17	5/49	14/26
21	chr4:65497558:A:C	TP	547.77	0/1	./.	0/16	12/12	0/37	14/39

Table 3: Manual review of small variant calling *(continued)*

Ex	Location	Manual assessment	Quality	LR genotype	GIAB genotype	Supporting reads/ Total reads Hap1	Supporting reads/ Total reads Hap2	Supporting reads/ Total reads PB	Supporting reads/ Total reads TruSeq
22	chr5:8079967:A:G	?	1174	0/1	1/1	~200/202	~200/200	42/47	28/28
23	chr5:72174473:G:A	FP	67.77	0/1	./.	1/10	4/16	0/42	0/23
24	chr6:19909664:A:C	FP	367.77	0/1	./.	0/22	0/10	0/67	0/24
25	chr7:2406995:A:C	FP	45.77	0/1	./.	0/7	0/5	1/38	3/27

Table 3: Manual review of small variant calling. Linked-Reads alignments made with Long Ranger using GATK, PacBio alignments and PCR-free TruSeq alignments made with BWA were loaded to IGV for manual inspection. Columns are: 1) Number; 2) Location; 3) Manual assessment of call; 4) Quality score of variant call from the VCF; 5) Long Ranger called genotype; 6) GIAB called genotype; 7) Read support for call in Haplotype 1; 8) Read support for call in Haploytpe 2; 9) Read support for call in PacBio; 10) Read support for call in PCR-free TruSeq; 11) File with screenshot of review.

Table 4: Summary of phasing accuracy analysis for lrWGS control samples

Sample	Phase Block N50 (bp)	Long Switch Error Rate	Short Switch Error Rate	Fraction Correct in Phase Block	Fraction Correct in Gene
NA12878	9424660	0	0	0.986	0.999
NA12878	6539869	0	0	0.976	1.000

Table 5: Gene, variant distance, and RVSI score for clinically-relevant genes

Sample	Gene	Var1	Var2	Variant distance	RVIS score	RVIS percent	Molecule length	Variant phased
B12-38	DYSF	chr2:71,778,243dupT	chr2:71,817,342_71,817,343delinsAA	39,097 bp	-1.31	4.65%	13,553 bp	No
B12-38	DYSF	chr2:71,778,243dupT	chr2:71,817,342_71,817,343delinsAA	39,097 bp	-1.31	4.65%	16,911 bp	No
B12-38	DYSF	chr2:71,778,243dupT	chr2:71,817,342_71,817,343delinsAA	39,097 bp	-1.31	4.65%	18,439 bp	No
B12-38	DYSF	chr2:71,778,243dupT	chr2:71,817,342_71,817,343delinsAA	39,097 bp	-1.31	4.65%	18,461 bp	Yes
B12-38	DYSF	chr2:71,778,243dupT	chr2:71,817,342_71,817,343delinsAA	39,097 bp	-1.31	4.65%	19,309 bp	Yes
B12-38	DYSF	chr2:71,778,243dupT	chr2:71,817,342_71,817,343delinsAA	39,097 bp	-1.31	4.65%	21,226 bp	Yes
B12-38	DYSF	chr2:71,778,243dupT	chr2:71,817,342_71,817,343delinsAA	39,097 bp	-1.31	4.65%	34,800 bp	Yes
B12-38	DYSF	chr2:71,778,243dupT	chr2:71,817,342_71,817,343delinsAA	39,097 bp	-1.31	4.65%	42,939 bp	Yes
B12-38	DYSF	chr2:71,778,243dupT	chr2:71,817,342_71,817,343delinsAA	39,097 bp	-1.31	4.65%	85,077 bp	Yes
B12-38	DYSF	chr2:71,778,243dupT	chr2:71,817,342_71,817,343delinsAA	39,097 bp	-1.31	4.65%	88,410 bp	Yes
B12-38	DYSF	chr2:71,778,243dupT	chr2:71,817,342_71,817,343delinsAA	39,097 bp	-1.31	4.65%	119,747 bp	Yes
B12-38	DYSF	chr2:71,778,243dupT	chr2:71,817,342_71,817,343delinsAA	39,097 bp	-1.31	4.65%	130,101 bp	Yes
B12-112	POMT2	chr14:77,745,107A>G	chr14:77,778,305C>T	33,198 bp	-0.93	9.68%	10,609 bp	No
B12-112	POMT2	chr14:77,745,107A>G	chr14:77,778,305C>T	33,198 bp	-0.93	9.68%	12277 bp	No
B12-112	POMT2	chr14:77,745,107A>G	chr14:77,778,305C>T	33,198 bp	-0.93	9.68%	15,536 bp	No
B12-112	POMT2	chr14:77,745,107A>G	chr14:77,778,305C>T	33,198 bp	-0.93	9.68%	16,546 bp	No
B12-112	POMT2	chr14:77,745,107A>G	chr14:77,778,305C>T	33,198 bp	-0.93	9.68%	20,782 bp	No
B12-112	POMT2	chr14:77,745,107A>G	chr14:77,778,305C>T	33,198 bp	-0.93	9.68%	21,106 bp	No
B12-112	POMT2	chr14:77,745,107A>G	chr14:77,778,305C>T	33,198 bp	-0.93	9.68%	21,858 bp	No
B12-112	POMT2	chr14:77,745,107A>G	chr14:77,778,305C>T	33,198 bp	-0.93	9.68%	54,569 bp	Yes
B12-112	POMT2	chr14:77,745,107A>G	chr14:77,778,305C>T	33,198 bp	-0.93	9.68%	55,546 bp	Yes
B12-112	POMT2	chr14:77,745,107A>G	chr14:77,778,305C>T	33,198 bp	-0.93	9.68%	107,082 bp	Yes

Table 5: Gene, variant distance, and RVSI score for clinically-relevant genes *(continued)*

Sample	Gene	Var1	Var2	Variant distance	RVIS score	RVIS percent	Molecule length	Variant phased
B12-112	POMT2	chr14:77,745,107A>G	chr14:77,778,305C>T	33,198 bp	-0.93	9.68%	112,692 bp	Yes
B12-21	TTN	chr2:179,585,773C>A	chr2:179,531,966C>A	53,807 bp	2.17	98.04%	17,432 bp	Yes
B12-21	TTN	chr2:179,585,773C>A	chr2:179,531,966C>A	53,807 bp	2.17	98.04%	18,128 bp	Yes
B12-21	TTN	chr2:179,585,773C>A	chr2:179,531,966C>A	53,807 bp	2.17	98.04%	18,158 bp	Yes
B12-21	TTN	chr2:179,585,773C>A	chr2:179,531,966C>A	53,807 bp	2.17	98.04%	20,756 bp	Yes
B12-21	TTN	chr2:179,585,773C>A	chr2:179,531,966C>A	53,807 bp	2.17	98.04%	28,799 bp	Yes
B12-21	TTN	chr2:179,585,773C>A	chr2:179,531,966C>A	53,807 bp	2.17	98.04%	29,796 bp	Yes
B12-21	TTN	chr2:179,585,773C>A	chr2:179,531,966C>A	53,807 bp	2.17	98.04%	47,443 bp	Yes
B12-21	TTN	chr2:179,585,773C>A	chr2:179,531,966C>A	53,807 bp	2.17	98.04%	63,218 bp	Yes
B12-21	TTN	chr2:179,585,773C>A	chr2:179,531,966C>A	53,807 bp	2.17	98.04%	64,199 bp	Yes
B12-21	TTN	chr2:179,585,773C>A	chr2:179,531,966C>A	53,807 bp	2.17	98.04%	67,034 bp	Yes
B12-21	TTN	chr2:179,585,773C>A	chr2:179,531,966C>A	53,807 bp	2.17	98.04%	90,767 bp	Yes
B12-21	TTN	chr2:179,585,773C>A	chr2:179,531,966C>A	53,807 bp	2.17	98.04%	93,253 bp	Yes
UC-394	TTN	chr2:179,584,098C>T	chr2:179,395,221T>A	188,877 bp	2.17	98.04%	13,118 bp	Yes
UC-394	TTN	chr2:179,584,098C>T	chr2:179,395,221T>A	188,877 bp	2.17	98.04%	16,791 bp	No
UC-394	TTN	chr2:179,584,098C>T	chr2:179,395,221T>A	188,877 bp	2.17	98.04%	18,192 bp	No
UC-394	TTN	chr2:179,584,098C>T	chr2:179,395,221T>A	188,877 bp	2.17	98.04%	18,841 bp	No
UC-394	TTN	chr2:179,584,098C>T	chr2:179,395,221T>A	188,877 bp	2.17	98.04%	28,033 bp	No
UC-394	TTN	chr2:179,584,098C>T	chr2:179,395,221T>A	188,877 bp	2.17	98.04%	30,653 bp	No
UC-394	TTN	chr2:179,584,098C>T	chr2:179,395,221T>A	188,877 bp	2.17	98.04%	32,530 bp	No
UC-394	TTN	chr2:179,584,098C>T	chr2:179,395,221T>A	188,877 bp	2.17	98.04%	69,939 bp	Yes
UC-394	TTN	chr2:179,584,098C>T	chr2:179,395,221T>A	188,877 bp	2.17	98.04%	87,045 bp	Yes

Table 5: Gene, variant distance, and RVSI score for clinically-relevant genes *(continued)*

Sample	Gene	Var1	Var2	Variant distance	RVIS score	RVIS percent	Molecule length	Variant phased
UC-394	TTN	chr2:179,584,098C>T	chr2:179,395,221T>A	188,877 bp	2.17	98.04%	88,605 bp	Yes
UC-394	TTN	chr2:179,584,098C>T	chr2:179,395,221T>A	188,877 bp	2.17	98.04%	89,863 bp	Yes

Table 5: Gene, variant distance, and RVSI score for clinically-relevant genes. Impact of molecule length and constraint on the ability of Linked-Reads to phase causative variants. As molecule length increases within a sample, the likelihood that two causative variants will be phased relative to each other also increases. However, genes that are not highly constrained (e.g. *TTN*) are more likely to show phasing between distant variants at small molecule lengths because more heterozygous variants are likely to occur between those variants than in highly constrained genes.

Table 6: Structural variant calls

	NA12878 lrWGS (ALL)	NA12878 lrWGS (PASS)	SVClassify	PacBio MtSinai (ALL)	PacBio MtSinai (PASS)	PacBio NGMLR (PASS)
total SVs	9923	4573	2676	38839	10310	22877
total DEL >30Kb	293	17	11	101	23	36
total DEL <30Kb	4569	4512	2665	20856	4472	9897
total INS >30Kb	NA	NA	NA	14	6	NA
total INS <30Kb	NA	NA	NA	17868	5809	12052
total INV >30Kb	2287	4	NA	NA	NA	57
total INV <30Kb	0	0	NA	NA	NA	93
total DUP >30Kb	288	6	NA	NA	NA	9
total DUP <30Kb	0	0	NA	NA	NA	594
total UNK >30Kb	1078	2	NA	NA	NA	NA
total UNK <30Kb	0	0	NA	NA	NA	NA
total DISTAL >30Kb	1380	8	NA	NA	NA	NA
total DISTAL <30Kb	28	24	NA	NA	NA	NA
total TRA >30Kb	NA	NA	NA	NA	NA	119
total DUP/INS <30Kb	NA	NA	NA	NA	NA	8
total INVDUP <30Kb	NA	NA	NA	NA	NA	12
total INV/INVDUP <30Kb	NA	NA	NA	NA	NA	NA
total DEL/INS <30Kb	NA	NA	NA	NA	NA	NA

Table 6: Structural variant calls and ground truth. Columns correspond to datasets generated for this article or by other groups that can be used as ground truth. The row segmentation corresponds to SVs larger or equal to 30Kb and smalled than that size. This segmentation correponds to the SVs reported in Long Ranger's large_svs.vcf and dels.vcf, respectively.

Table 7: Mendelian analysis

Locus	NA12878	NA12892	NA12891	In svclassify?	Classification	Description
chr1:72766325-72811837	1|1	1|1	1|1	Yes	TP	NA
chr1:152555548-152587734	0|1	-	1|1	Yes	TP	NA
chr2:34695837-34736559	1|0	0|1	1|1	Yes	TP	NA
chr2:52749692-52785263	1|1	0|1	1|1	Yes	TP	NA
chr3:129763385-129806737	1|1	1|1	1|0	Yes	TP	NA
chr3:162512134-162626333	1|0	0|1	1|1	Yes	TP	NA
chr4:34779956-34828940	0|1	0|1	0|1	Yes	TP	NA
chr5:104432114-104503672	1|0	1|0	-	Yes	TP	NA
chr1:189690000-189790000	0/1	1|0	-	No	likely-FP	Deletion super-setting the loci below. Breakpoint disagreement with mother (chr1:189704514-189783350)
chr1:189704517-189783347	1|0	1|0	-	No	TP	Breakpoint agreement with mother (chr1:189704514-189783350)
chr11:55360000-55490000	0/1	-	0/1	No	Complex	Different 3' breakpoint with father (chr11:55360000-55430000), Overlaps inheritance consistent UNK call
chr2:242900000-243080000	0/1	-	-	No	likely-FP	Missing inheritance support
chr20:1561086-1594155	0|1	1|1	-	No	TP	NA
chr4:161043706-161074850	1|0	-	1|0	No	TP	NA
chr6:67008738-67048908	1|0	1|0	-	No	TP	NA
chr6:78967204-79036470	0|1	-	0|1	No	TP	NA
chr8:39232084-39387222	1|0	1|0	-	No	TP	NA

Table 7: Description of structural variant calls unique to svclassify or Long Ranger. Calls were compared to Long Ranger calls made in NA12878, NA12892, NA12891 and manually reviewed.

Table 8: Coriell samples

X1	X2	Samples	Event in Sample	Barcode Coverage	Barcode Overlap	Both Methods
Copy Number Losses	Terminal Events	GM06936: 46,XX,del(10)(:p13>qter)	Deletion	Yes	No†	Yes
.	.	GM10989: 46,XY,del(9)(p23)	Deletion	Yes	No†	Yes
.	.	GM20027: 45,X	Aneuploidy	Yes	No†	Yes
.	.	GM21886: 46,XY,r(18)(p11q21)	Ring chromosome	Yes	No†	Yes
.	.	GM06226*: 46,XY,der(1)t(1;16)(q44;p12)mat	Derivative chromosome	Yes	No†	Yes
.	.	GM21699*: 46,XY,der(6)t(3;6)(p26;q26)	Derivative chromosome	Yes	No†	Yes
.	.	GM14485*: 46,XY,der(8)del(8)(p23.3)dup(8)(:p23.1->p11.2::p23.1->qter)	Derivative chromosome	Yes	No†	Yes
Copy Number Losses	Non-Terminal Events	GM09888: 46,XX,del(8)(q23q24.1)	Deletion	Yes	Yes	Yes
.	.	GM14164: 46,XX,del(13)(q13q32)	Deletion	Yes	Yes	Yes
.	.	GM09216: 46,XY,del(2)(p25.1p23)	Deletion	Yes	Yes	Yes
.	.	GM10925‡: 46,XY,del(7)(p14p12)	Deletion	No	Yes	Yes
Copy Number Gains	Terminal Events	GM05966: 46,XY,dup(14)(pter->q24::q22->qter)	Duplication	Yes	No†	Yes
.	.	GM01416: 48,XXXX	Aneuploidy	Yes	No†	Yes
.	.	GM05067: 47,XY,+del(9)(q11)mat	Partial Aneuploidy	Yes	No†	Yes
.	.	GM16362: 47,XY,+del(22)(q11.2q13.3)	Partial Aneuploidy	Yes	No†	Yes
.	.	GM20556: 47,XY,+idic(15)(q13)	Isodicentric chromosome	Yes	No†	Yes
.	.	GM06870: 47,XX,+i(18)(p10)	Isodicentric chromosome	Yes	No†	Yes

Table 8: Coriell samples *(continued)*

X1	X2	Samples	Event in Sample	Barcode Coverage	Barcode Overlap	Both Methods
.	.	GM06226*: 46,XY,der(1)t(1;16)(q44;p12)mat	Derivative chromosome	Yes	No†	Yes
.	.	GM21699*: 46,XY,der(6)t(3;6)(p26;q26)	Derivative chromosome	Yes	No†	Yes
.	.	GM14485*: 46,XY,der(8)del(8)(p23.3)dup(8)(:p23.1->p11.2::p23.1->qter)	Derivative chromosome	Yes	No†	Yes
Copy Number Gains	Non-Terminal Events	GM09367: 46,XX,dup(6)(q21q24)	Duplication	Yes	Yes	Yes
Copy Neutral Events	Translocations	GM06226*: 46,XY,der(1)t(1;16)(q44;p12)mat	Derivative chromosome	No†	Yes	Yes
.	.	GM21699*: 46,XY,der(6)t(3;6)(p26;q26)	Derivative chromosome	No†	Yes	Yes
.	.	GM14485*: 46,XY,der(8)del(8)(p23.3)dup(8)(:p23.1->p11.2::p23.1->qter)	Derivative chromosome	No†	Yes	Yes
.	.	GM22765: 46,XY,t(4;14;11)(q34.1;q21;q22.2)	Balanced translocation	No†	Yes	Yes
.	.	GM10207: 46,XY,t(10;14)(10qter>10p13::14q24.3>14qter;14pter>14q24.3::10p13>10pter)	Balanced translocation	No†	Yes	Yes
.	.	GM18825: 46,XX,t(5;10)(p13.3;q21.1)	Balanced translocation	No†	Yes	Yes
.	.	GM22709§: 46,XY,t(16;20)(q11.2;q13.2)	Balanced translocation	No†	No	No
Copy Neutral Events	Inversions	GM21075: 46,XY,inv(9)(q22.3q34.1)	Inversion	No†	Yes	Yes

Table 8: Long Ranger SV analysis of 23 Coriell samples with multiply-confirmed balanced or unbalanced SVs. *Sample contains multiple structural variants. †Algorithm not expected to detect this variant type. ‡Deletion in GM10925 falls in a segmental duplication; was called with high-quality score by Long Ranger but filtered as a likely false positive. §Balanced translocation in GM22709 falls within a heterochromatic region on chromosome 16 where there are known gaps in the reference assembly.

Table 9: Intermediate SV calls with other call sets

Intermediate SV metrics	NA12878
Number of deletion calls from GATK	1,824
Number of deletion calls from LongRanger	4,118
Number of merged calls	5,136
Average deletion size	696bp
Number of heterozygous calls	3,015
Number of homozygous calls	2,038
Number of svclassify merged calls	5,390
Number of calls that match Svclassify truth set (Recall)	2,024 (88.2%)
Number of false positive calls (Precision)	3,109 (39.4%)
Number of false negative calls	257
Comparison to Lumpy	NA12878
Number of deletion calls	19,307
Number of svclassify merged calls	10,588
Average deletion size	767bp
Number of calls that match Svclassify truth set (Recall)	1,263 (55.4%)
Number of false positive calls (Precision)	8,307 (13.2%)
Number of false negative calls	1018

Table 9: Extended intermediate SV (50 bp to 30 kb) results. Long Ranger produces SV calls in the 50 bp to 30 kb range using barcode-related algorithms described above. Additionally, small indels are called using the Genome Analysis Toolkit (GATK). These two approaches work synergistically, with GATK's ability to call indels falling off as a function of read length (in this case, 2X150 bp). To evaluate this, we used SURVIVOR (Jeffares et al. 2017) to merge deletions >=50 bp called by GATK with the intermediate SVs called by Long Ranger. This merged variant set was then merged again with SURVIVOR with the svclassify truth set (Parikh et al. 2016) in order to report the resulting true positive and false positive rates as well as the associated recall and precision. This is the same as Table 4 in the main text, but with the input being the merged results of Long Ranger deletions

and GATK instead of just Long Ranger. We saw that the addition of GATK added only 7 new true positive hits, reflecting the lack of small variants in the svclassify truth set.

To establish a comparison to existing methods, we also ran the Long Ranger alignments through the lumpyexpress (Layer et al., 2014) structural variant calling tool using standard parameters. We found that lumpyexpress called more than three times as many variants, with lower true positive and much higher false positive rates.

Supplemental Files

Supplemental File 1: Per variant report of PacBio evidence for putative false positive calls.
